# Metabolomic phenotyping of obesity for profiling cardiovascular and ocular diseases

**DOI:** 10.1186/s12967-023-04244-x

**Published:** 2023-06-12

**Authors:** Pingting Zhong, Shaoying Tan, Zhuoting Zhu, Gabriella Bulloch, Erping Long, Wenyong Huang, Mingguang He, Wei Wang

**Affiliations:** 1 State Key Laboratory of Ophthalmology, Zhongshan Ophthalmic Center, Sun Yat-Sen University, Guangdong Provincial Key Laboratory of Ophthalmology and Visual Science, Guangdong Provincial Clinical Research Center for Ocular Diseases, Guangzhou, China; 2 School of Optometry, The Hong Kong Polytechnic University, Hong Kong, China; 3 Research Centre for SHARP Vision, The Hong Kong Polytechnic University, Hong Kong, China; 4 Centre for Eye and Vision Research (CEVR), 17W Hong Kong Science Park, Hong Kong, China; 5 Centre for Eye Research Australia, Royal Victorian Eye and Ear Hospital, University of Melbourne, Level 7, 32 Gisborne Street, Melbourne, VIC 3002 Australia; 6 Department of Ecology and Evolutionary Biology, University of Michigan, Ann Arbor, MI 48109 USA

**Keywords:** Metabolome, NMR, Obesity, Mortality, Systemic diseases, Age-related eye disease, Comorbidity

## Abstract

**Background:**

We aimed to evaluate the impacts of metabolomic body mass index (metBMI) phenotypes on the risks of cardiovascular and ocular diseases outcomes.

**Methods:**

This study included cohorts in UK and Guangzhou, China. By leveraging the serum metabolome and BMI data from UK Biobank, this study developed and validated a metBMI prediction model using a ridge regression model among 89,830 participants based on 249 metabolites. Five obesity phenotypes were obtained by metBMI and actual BMI (actBMI): normal weight (NW, metBMI of 18.5–24.9 kg/m^2^), overweight (OW, metBMI of 25–29.9 kg/m^2^), obesity (OB, metBMI ≥ 30 kg/m^2^), overestimated (OE, metBMI-actBMI > 5 kg/m^2^), and underestimated (UE, metBMI-actBMI < − 5 kg/m^2^). Additional participants from the Guangzhou Diabetes Eye Study (GDES) were included for validating the hypothesis. Outcomes included all-cause and cardiovascular (CVD)-cause mortality, as well as incident CVD (coronary heart disease, heart failure, myocardial infarction [MI], and stroke) and age-related eye diseases (age-related macular degeneration [AMD], cataracts, glaucoma, and diabetic retinopathy [DR]).

**Results:**

In the UKB, although OE group had lower actBMI than NW group, the OE group had a significantly higher risk of all-cause mortality than those in NW prediction group (HR, 1.68; 95% CI 1.16–2.43). Similarly, the OE group had a 1.7–3.6-fold higher risk than their NW counterparts for cardiovascular mortality, heart failure, myocardial infarction, and coronary heart disease (all P < 0.05). In addition, risk of age-related macular denegation (HR, 1.96; 95% CI 1.02–3.77) was significantly higher in OE group. In the contrast, UE and OB groups showed similar risks of mortality and of cardiovascular and age-related eye diseases (all P > 0.05), though the UE group had significantly higher actBMI than OB group. In the GDES cohort, we further confirmed the potential of metabolic BMI (metBMI) fingerprints for risk stratification of cardiovascular diseases using a different metabolomic approach.

**Conclusions:**

Gaps of metBMI and actBMI identified novel metabolic subtypes, which exhibit distinctive cardiovascular and ocular risk profiles. The groups carrying obesity-related metabolites were at higher risk of mortality and morbidity than those with normal health metabolites. Metabolomics allowed for leveraging the future of diagnosis and management of ‘healthily obese’ and ‘unhealthily lean’ individuals.

**Supplementary Information:**

The online version contains supplementary material available at 10.1186/s12967-023-04244-x.

## Introduction

The obesity epidemic is responsible for 8.5 percent of global deaths and is associated with an increased risk of multiple chronic diseases, including cardiovascular diseases (CVD), type 2 diabetes mellitus (T2DM), and age-related eye disease [1, 2]. As the most commonly used indicator for obesity, the body mass index (BMI) can hardly explain the relationship between obese anthropometrics and multi-diseases [3]. This has led to the ‘obesity paradox’, whereby up to 20 percent of patients with T2DM have a normal BMI, and those with obese BMIs never develop T2DM [4, 5]. Similar phenomena have been noted for CVD and other diseases associated with poor lifestyle [6].

Metabolomic profiling had potential for redefining obesity, considering the added value of serum metabolites for predicting T2DM, CVD, and mortality independent of BMI [7–9]. This notion had been strengthened by Cirulli et al. [10], who recently introduced the gap between metabolome-predicted BMI (metBMI) and actual BMI (actBMI) as an indicator for a more precise and detailed phenotyping of obesity. Ottosson et al. [11] subsequently demonstrated that the ‘unhealthily lean’ individuals, defined by overestimated metBMI, had significant higher risk for T2DM and mortality compared with their metabolically healthy, normal metBMI counterparts. Despite these recent insights, the impacts of metabolomic-defined phenotypes on future CVD and ocular outcomes are unknown [2, 12, 13].

We hypothesise that metabolically unhealthy phenotype had greater risk of death, CVD, and major eye diseases than metabolically healthy phenotype. The largest UK Biobank (UKB) metabolome study offers a unique opportunity to affirm this hypothesis by utilising data metabolomic data from over 0.1 million individuals [14]. This study aims to investigate the risk of major diseases associated with metabolic obesity in different metBMI categories, providing insights into the metabolomic signature of obesity and its implications for personalized prevention. To ensure robust findings, we tested the potential of metBMI fingerprints in an independent Chinese cohort, utilizing a distinct metabolomic approach.

## Methods

### Design and population

**Fig. 1:**
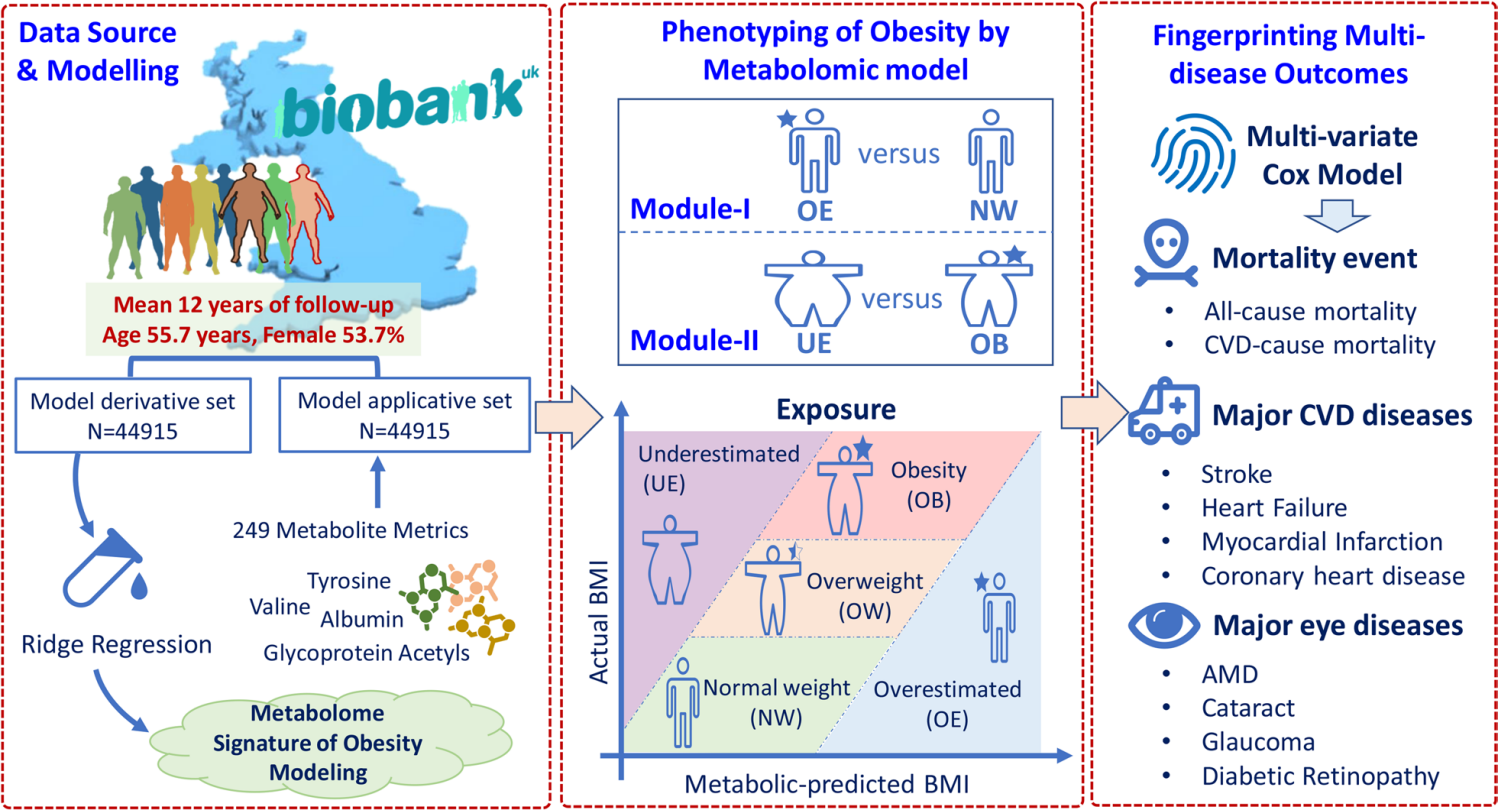
Overall workflow of the study. Five categories were obtained by difference of the metabolome-defined BMI (metBMI) and actual BMI (actBMI). BMI: body mass index; metBMI: metabolic body mass index; CVD: cardiovascular disease; NW: normal weight of metabolic BMI; OW: overweight of metabolic BMI; OB: obesity of metabolic BMI; OE: metabolome-defined BMI over than actual BMI; UE: metabolome-defined BMI under than actual BMI; AMD: age-related macular degeneration

**Fig. 2:**
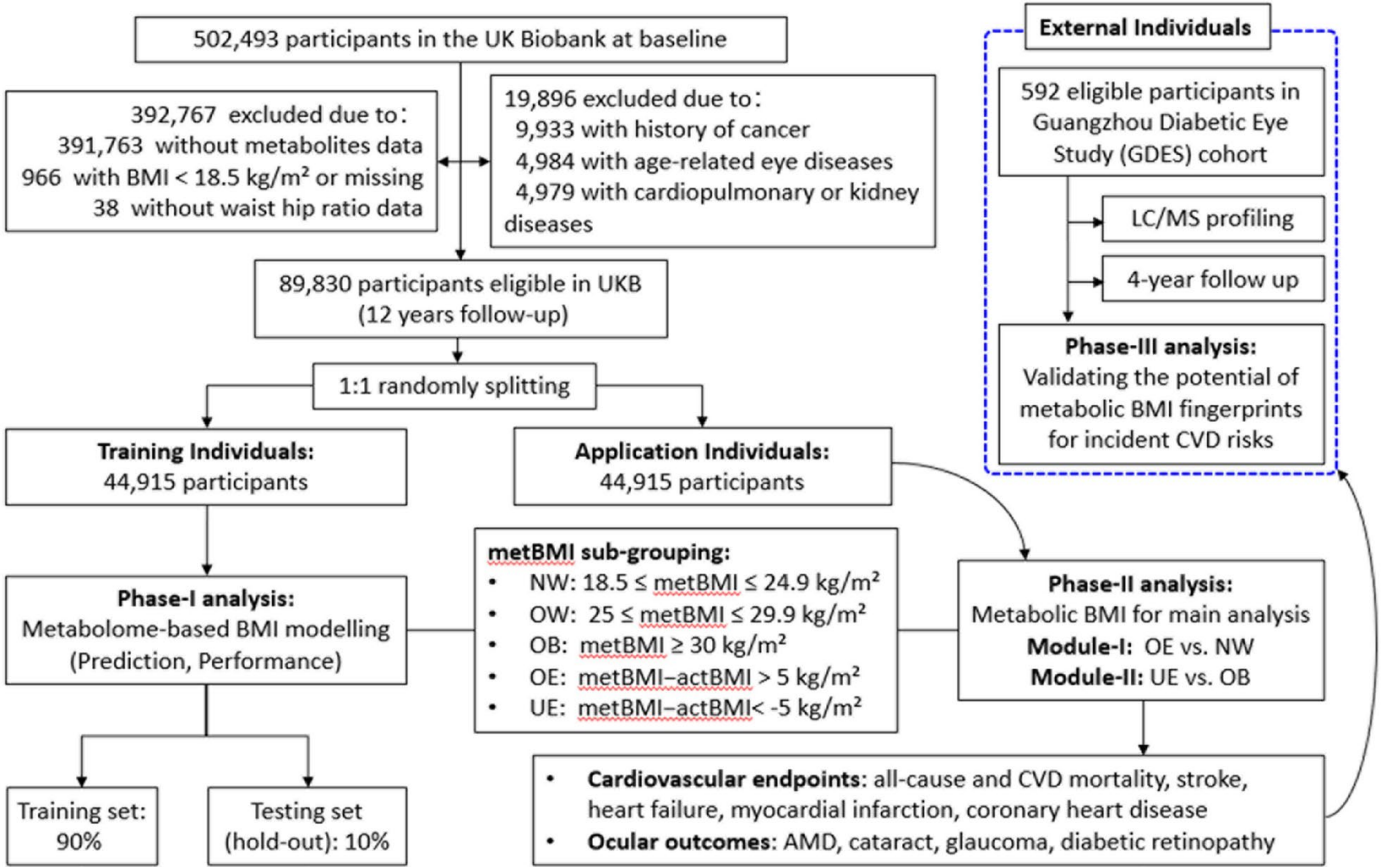
Flowchart for selecting participants in the whole study. BMI: body mass index; metBMI: metabolic body mass index; CVD: cardiovascular disease; NW: normal weight of metabolic BMI; OW: overweight of metabolic BMI; OB: obesity of metabolic BMI; OE: metabolome-defined BMI over than actual BMI; UE: metabolome-defined BMI under than actual BMI; AMD: age-related macular degeneration

Figure 1 shows the overall design of this study with UKB data. The UKB recruited more than 0.5 million individuals aged 40 to 69 years from 22 assessment centres in the UK between 2006 and 2010 [15]. The use of data for this analysis was approved by the UKB committee (Approved Research ID: 62443). Participants with adequate anthropometric and metabolomic data were eligible for this study. The participants with a BMI < 18.5 kg/m^2^, a history of CVD or age-related eye diseases at baseline were excluded (Fig. 2). Then the eligible participants, with 12 years of follow-up, were randomly divided into a derivative set and an applicative set. In the phase-I, a Ridge regression model was created to develop the metabolic BMI (metBMI) model to predict metBMI. In the phase-II, the metBMI in the applicative set was used and categorised as metBMI subgroups for the assessment of incident cardiovascular and ocular diseases between the groups matched or mismatched the metBMI prediction error margin. For the phase-III analysis, data from the Guangzhou Diabetes Eye Study (GDES) was incorporated as an external cohort. GDES is a community-based study that recruited 2300 participants with type 2 diabetes (T2D) aged 35–85 between 2017 and 2019 in Guangzhou, China. Participants with available data on both BMI and metabolome were included in the analysis (Fig. 2). This study was conducted with the approval of the Ethics Committee of Zhongshan Ophthalmic Center (2017KYPJ094).

### Metabolome profiling in UKB

At baseline, serum samples were collected and subjected to nuclear magnetic resonance (NMR) based metabolome profiling (Nightingale Health, Helsinki, Finland). A detailed description of the experimental protocol has been documented elsewhere [14, 16]. In total, 110,730 participants with 249 metabolomic biomarkers data were available at baseline, of which the panel was grouped into 14 subclasses according to metabolite class. There were 249 metabolomic metrics (168 measured biomarkers and 81 ratio parameters) (Additional file 1: Tables S1 and S2). All serum metabolite levels were pre-processed using natural log transformation (ln[X + 1]) followed by Z normalisation (Additional file 1: Method S1).

### Definitions of exposures

The actBMI was calculated as weight in kilograms divided by height squared, according to the World Health Organization (WHO) definition, then categorised as normal weight (18.5–24.9 kg/m^2^), overweight (25–29.9 kg/m^2^), or obese (≥ 30.0 kg/m^2^) [17]. According to reports by Cirulli et al. [10] and Ottosson et al. [11], the gap of metBMI and actBMI had a normal estimation within a margin of error of 5 kg/m^2^ (metBMI − actBMI range of − 5 to + 5 kg/m^2^), which was used to define the subtypes of obesity. Five categories were obtained to describe metBMI subgroups: normal weight (NW, metBMI, 18.5–24.9 kg/m^2^), overweight (OW, metBMI, 25–29.9 kg/m^2^), and obesity (OB, metBMI ≥ 30 kg/m^2^), overestimated (OE, metBMI − actBMI > 5 kg/m^2^) or underestimated (UE, metBMI − actBMI < -5 kg/m^2^). Two comparison groups (Module-I: OE vs. NW; Module-II: UE vs. OB) were obtained (Figs. 1 & 2).

### Definitions of endpoints

For UKB, the main outcomes were all-cause and CVD-cause mortality, as well as incident CVD and age-related eye diseases (Fig. 1). Incident CVD included stroke, heart failure, myocardial infarction, and coronary heart disease. Age-related eye diseases included age-related macular degeneration (AMD), cataract, glaucoma, and diabetic retinopathy (DR). The ICD-10 and OPCS4 codes for each outcome are listed in Additional file 1: Table S3.

### Assessment of covariates

A detailed questionnaire collected information on age, sex, and ethnicity, educational attainment, Townsend deprivation index scores (a higher index score indicated lower regional socioeconomic status), lifestyle information on smoking and drinking habits, physical activity (metabolically equivalent hours of physical activity per week during work and leisure time), and healthy diet scores. A healthy diet score was a part of on Bradbury et al.’s healthy lifestyle factor score and it was calculated based on consumption of commonly eaten food groups (Fruits: ≥ 3 servings/day, Vegetables: ≥ 3 servings/day, Fish: ≥ 2 servings/week, Processed meats: ≤ 1 serving/week, Unprocessed red meats: ≤ 1.5 servings/week, Whole grains: ≥ 3 servings/day, Refined grains: ≤ 1.5 servings/day) [18]. Detailed assessment of diet is shown in Additional file 1: Method S2. A healthy dietary pattern is defined as the consumption of at least four out of the seven commonly consumed food groups, in line with recommendations for prioritizing cardiometabolic health [19]. The total cholesterol, high-density lipoprotein (HDL), glycated haemoglobin A1c (HbA1c), and serum creatinine levels were determined by standard blood tests. According to the American Diabetes Association’s criteria, participants with prediabetes were defined as having HbA1c of 5.7–6.4% (39–47 mmol/mol), while diabetes was defined as self-reported or physician-diagnosed diabetes, with the use of antihyperglycemic medications, or with an HbA1c level ≥ 6.5 percent.

### External validation of the hypothesis in GDES

This study included a total of 592 participants from the GDES cohort who met similar eligibility criteria as those from the UKB for analysis (Fig. 2). At baseline, all participants underwent Liquid Chromatography Tandem Triple Quadrupole Mass Spectrometry (LC, ExionLC AD, SCIEX, USA; MS, QTRAP® System, SCIEX, USA) for metabolomics profiling (Additional file 1: Method S3). Incident CVD was defined as the occurrence of coronary heart disease, heart failure, atrial fibrillation, stroke, or related mortality during the follow-up period. This was determined by a combination of medical records, standard questionnaires, and verbal interviews.

### Statistical analyses

Data have been expressed as means (standard deviation, SD) and medians (interquartile range, IQR) for quantitative variables with normal and abnormal distributions, respectively. Numbers (percentage) presented categorical variables for the risk factors among the study participants. The chi-squared or independent t-test was used to compare the baseline characteristics across groups, where appropriate.

For the UKB, in the phase-I, to develop and validate the metabolomic BMI prediction model, eligible participants were randomly divided into derivative and applicative sets (1:1 split). To address multicollinearity among the metabolites and to obtain predictions for all participants, the ridge regression model (alpha value set to zero) incorporated 249 metabolomic metrics, in Stata software, was adopted for modelling the predictive algorithm according to previous reported methods by Cirulli et al. [10] and Ottosson et al. [11]. The tenfold cross-validation was used for all nonzero coefficients in the derivative set. Consequently, this procedure generated ten fitted sparse models and one single testing (hold-out) set-derived prediction for each participant. The optimal lambda value was 0.180 (Additional file 1: Method S4). The performance of the model was evaluated using the R-squared (r^2^) and the area under the curve (AUC). In the phase-II, the predictive model was then applied into applicative population, and the derived metBMI was used for subsequent analysis.

The individuals in the applicative set were categorized into five subtypes according to the difference between the metBMI and actBMI. Two analyses were performed: module-I compared the OE vs. NW groups, and module-II compared the UE vs. OB groups (Fig. 1). The survival curves were constructed and log-rank test was used to compare the accumulated survival probability of mortality, CVD, and ocular diseases across groups. Two multivariate Cox models were constructed: Model 1 was adjusted for age, sex, ethnicity, and Townsend deprivation index. Model 2 was additionally adjusted for educational attainment; actBMI; systolic blood pressure; smoking consumption; alcohol consumption; physical activity; total cholesterol, HDL, HbA1c, and serum creatinine levels; antihypertensive medications; statin use; and healthy diet score. The degree of association was expressed as the risk ratio (hazard ratio [HR]) with a 95% confidence interval (95% CI).

To evaluate the robustness of the primary analyses, subgroup analysis according to sex was performed. Furthermore, we conducted healthy diet style and central obesity subgroup analyses to investigate whether the OE group was at a higher risk of endpoint events compared to the NW group. Additionally, receiver operating characteristic (ROC) curves were used to assess the combined risk factors based on metBMI, waist-to-hip ratio (WHR), and serum creatinine in distinguishing NW from other metBMI subgroups. Furthermore, we explored an alternative obesity indicator—WHR—and its metabolic performance in assessing the risk of cardiovascular and ocular health, using the same model scheme (Additional file 1: Method S4). All statistical analyses were performed using Stata version 17 (Stata Corp., USA) software. A two-sided P-value of < 0.05 was considered statistically significant.

## Results

### Baseline characteristics of the population

In the UKB, of the 89,830 eligible participants, the mean age (SD) was 55.7 (8.1) years; 48,192 (53.7%) of the participants were female (Additional file 1: Table S4). The eligible subjects were randomly divided into a derivative set (n = 44,915) and an applicative set (n = 44,915), with similar baseline characteristics between the datasets.

### Application of metBMI model in UKB

**Fig. 3:**
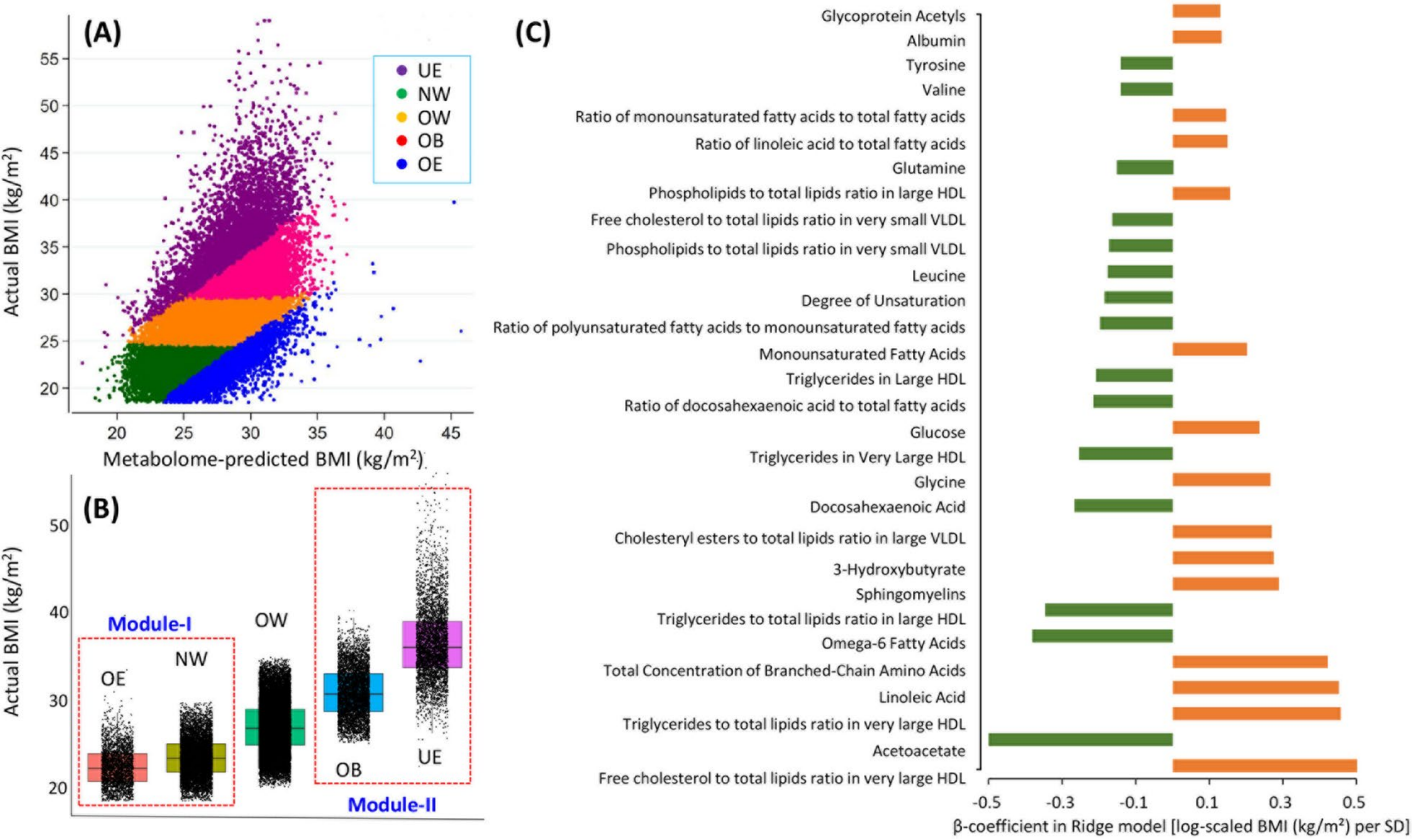
Distribution of metabolomic BMI, actual BMI, and metabolites used to predict BMI in the applicative set. **A** subgrouping for all individuals in application dataset, the correlation r^2^ value is 0.381; **B** distribution of actBMI across groups; **C** refers to the top 30 metabolites with high correlation ratio selected by ridge regression model. NW: normal weight of metabolic BMI; OW: overweight of metabolic BMI; OB: obesity of metabolic BMI; OE: metabolome-defined BMI over than actual BMI; UE: metabolome-defined BMI under than actual BMI

The ridge regression model performed excellently and explained nearly 40 percent of the variation in BMI in the training and application sets (r^2^ = 0.389 and 0.381, respectively). The model showed good performance in predicting BMI in the application set, with an area under the curve (AUC) of 0.799 (0.794–0.804). When applied the model to applicative set, five subtypes were obtained, with 7614 (16.9%), 25,574 (56.9%), 4921 (11.0%), 2631 (5.9%) and 4175 (9.3%) individuals classifying into the NW, OW, OB, OE, and UE groups, respectively (Fig. 3A, Additional file 1: Table S5). Although the actBMIs were normal in the OE (actBMI = 22.4 kg/m^2^) and NW (actBMI = 23.5 kg/m^2^) groups, the metBMI obtained from the OE group (metBMI = 28.8 kg/m^2^) were significantly higher than those from the NW group (metBMI = 23.7 kg/m^2^) (Table 1, Fig. 3B). Both the actBMIs and metBMIs of the UE and OB groups met or marginally reached the diagnostic criteria for obesity. Although the actBMIs of the UE group (actBMI = 36.6 kg/m^2^) were significantly higher than those of the OB group (actBMI = 30.9 kg/m^2^), the metBMIs of UE was lower than that in the OB (OB group: metBMI = 31.2 kg/m^2^; UE group: metBMI = 28.6 kg/m^2^) (Table 1, Fig. 3B). Top 30 metabolites that most strongly correlated with BMI prediction included glycoprotein acetyl, tyrosine, and valine, while albumin, glutamine, and leucine had the strongest negative correlations (Fig. 3C).

**Table 1:** Baseline characteristics of the included participants in the model applicative set

Characteristics	Participants in module-I			Participants in module-II		
	NW	OE	P-value	OB	UE	P-value
No. of subjects	7614	2631	–	4921	4175	–
Body mass index (BMI), kg/m^2^						
Actual BMI	23.5 ± 2.3	22.4 ± 2.2	< 0.001	30.9 ± 2.9	36.6 ± 4.4	< 0.001
Metabolomic-estimated	23.7 ± 1.0	28.8 ± 2.4	< 0.001	31.2 ± 1.0	28.6 ± 2.5	< 0.001
Waist hip ratio	0.79 ± 0.06	0.85 ± 0.08	< 0.001	0.94 ± 0.07	0.90 ± 0.09	< 0.001
Systolic blood pressure, mm Hg	130.1 ± 18.5	134.9 ± 18.7	< 0.001	143.3 ± 17.5	140.6 ± 17.6	< 0.001
Total cholesterol, mmol/L	5.64 (4.99–6.31)	5.53 (4.72–6.38)	< 0.001	5.50 (4.67–6.41)	5.58 (4.84–6.31)	0.038
High-density lipoprotein, mmol/L	1.8 ± 0.4	1.4 ± 0.3	< 0.001	1.1 ± 0.2	1.4 ± 0.4	< 0.001
Hemoglobin A1c (HbA1c), %	5.2 ± 0.4	5.5 ± 0.7	< 0.001	5.8 ± 1.0	5.6 ± 0.7	0.001
Serum creatinine, mg/dL	0.74 (0.66–0.84)	0.78 (0.67–0.90)	< 0.001	0.85 (0.73–0.96)	0.78 (0.68–0.89)	< 0.001
Presence of hypertension, %	4,054 (53.2%)	1,702 (64.7%)	< 0.001	4,306 (87.5%)	3,537 (84.7%)	< 0.001
Presence of diabetes mellitus, %	74 (1.0%)	152 (5.8%)	< 0.001	817 (16.6%)	472 (11.3%)	< 0.001
Healthy diet score	4.3 ± 1.3	3.6 ± 1.5	< 0.001	3.4 ± 1.4	3.8 ± 1.4	< 0.001

Data are presented as mean ± SD or No. (%). OE: metabolite-defined BMI over than actual BMI; NW: normal weight of metabolic BMI; OB: obesity of metabolic BMI; UE: metabolite-defined BMI under than actual BMI; BMI: body mass index. P-value was estimated based on chi-squared or independent t-test, where appropriate

### Baseline characteristics and incident events in the extreme subtypes in UKB

Serum total cholesterol levels were significantly higher and HDL levels were significantly lower in the OE group than in the NW group. Additionally, the OE group had significantly higher HbA1c levels and higher incidence rates of diabetes and hypertension than the NW prediction group (Table 1).

At the 12-year follow-up, 2233 (5.0%) all-cause and 535 (1.2%) CVD deaths had occurred in the applicative set. For CVD events, 469 (1.0%) participants were diagnosed with stroke, 900 (2.0%) with heart failure, 911 (2.0%) with myocardial infarction, and 3239 (7.2%) with coronary heart disease events. For age-related eye diseases, AMD occurred in 496 (1.1%) individuals, cataract in 3281 (7.3%), glaucoma in 808 (1.8%), and DR in 532 (1.2%) (Additional file 1: Table S5).

### Results of Cox regression analysis for applicative set in UKB

**Fig. 4:**
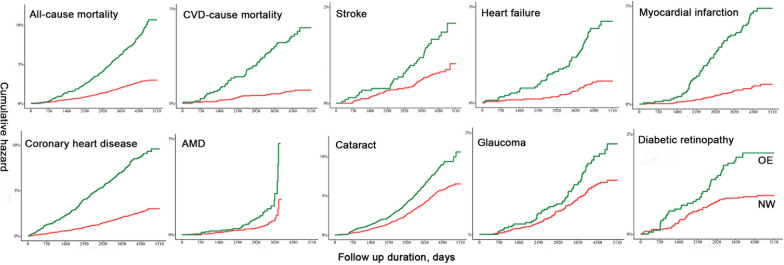
Survival plots showing the risks of cardiovascular and ocular diseases outcomes comparing OE versus NW groups. OE: metabolome-defined BMI over than actual BMI; NW: normal weight of metabolic BMI; CVD: cardiovascular disease; AMD: age-related macular degeneration. The log-rank tests for NW and OE group show: P-values are < 0.001 for all the incident events, except for incident AMD (P = 0.002) and glaucoma (P = 0.003)

During the mean 12-year follow-up, the OE group exhibited a significantly higher risk of most outcomes than the NW prediction group (Fig. 4). While no significant difference was found between OB and UE groups (Additional file 1: Fig. S1). After fully adjusted for other factors, the risks of all-cause mortality and CVD-mortality showed a 1.68 to 3.55-fold higher risk in the OE group than in the NW group (Table 2). For CVD events, the OE group was more likely to have heart failure (HR, 2.20; 95% CI 1.04–4.65; P = 0.040), myocardial infarction (HR, 2.47; 95% CI 1.18–5.17; P = 0.017), and coronary heart disease (HR, 1.72; 95% CI 1.20–2.46; P = 0.003). In addition, the OE group exhibited a 1.96-fold (95% CI 1.02–3.77) higher risk of AMD compared with their NW counterparts (Table 2).

**Table 2:** Associations of metabolome phenotypes with risks of cardiovascular and ocular disease outcomes in the applicative set

	Model 1*		Model 2^†^	
	HR (95% CI)	P	HR (95% CI)	P
Module-I (OE vs. NW)				
All-cause mortality	2.20 (1.81, 2.68)	< 0.001	1.68 (1.16, 2.43)	0.006
CVD-cause mortality	2.41 (2.16, 5.39)	< 0.001	3.55 (1.46, 8.64)	0.005
Stroke	1.48 (0.95, 2.30)	0.085	1.06 (0.48, 2.33)	0.889
Heart failure	2.47 (1.66, 3.68)	< 0.001	2.20 (1.04, 4.65)	0.040
Myocardial infarction	3.47 (2.32, 5.19)	< 0.001	2.47 (1.18, 5.17)	0.017
Coronary heart disease	2.32 (1.90, 2.83)	< 0.001	1.72 (1.20, 2.46)	0.003
AMD	1.68 (1.11, 2.53)	0.013	1.96 (1.02, 3.77)	0.044
Cataract	1.19 (1.00, 1.40)	0.045	1.11 (0.82, 1.50)	0.485
Glaucoma	1.20 (0.87, 1.66)	0.278	0.95 (0.55, 1.65)	0.865
Diabetic retinopathy	1.55 (1.03, 2.35)	0.037	1.36 (0.67, 2.77)	0.399
Module-II (UE vs. OB)				
All-cause mortality	0.91 (0.77, 1.08)	0.284	0.84 (0.62, 1.14)	0.258
CVD-cause mortality	1.13 (0.84, 1.53)	0.423	1.09 (0.62, 1.91)	0.771
Stroke	1.46 (1.02, 2.11)	0.004	1.40 (0.70, 2.82)	0.344
Heart failure	1.57 (1.26, 1.94)	< 0.001	1.21 (0.81, 1.80)	0.345
Myocardial infarction	0.84 (0.66, 1.07)	0.149	0.75 (0.49, 1.16)	0.200
Coronary heart disease	0.91 (0.80, 1.04)	0.169	0.96 (0.75, 1.23)	0.739
AMD	1.02 (0.69, 1.49)	0.938	0.68 (0.32, 1.41)	0.297
Cataract	0.82 (0.71, 0.95)	0.009	0.84 (0.63, 1.11)	0.220
Glaucoma	0.97 (0.70, 1.35)	0.875	1.46 (0.79, 2.70)	0.224
Diabetic retinopathy	1.06 (0.78, 1.44)	0.721	1.08 (0.60, 1.95)	0.793

BMI: body mass index; OE: metabolome-defined BMI over than actual BMI; NW: normal weight of metabolic BMI; UE: metabolome-defined BMI under than actual BMI; OB: obesity of metabolic BMI; CVD: cardiovascular diseases; AMD: age-related macular degeneration; HR: hazard ratio; CI: confidence interval

^*^Model 1: adjusted for age (continuous), sex (female and male), ethnicity (white/others), and Townsend deprivation index (continuous)

^†^Model 2: further adjusted for educational attainment (above or below college/university degree), actual body mass index (continuous), systolic blood pressure (continuous); smoking and alcohol drinking status (never/previous/present), physical activity (continuous), total cholesterol (continuous), high-density lipoprotein (continuous), hemoglobin A1c (continuous), serum creatinine (continuous), antihypertensive medications (yes/no), statin medications (yes/no), and healthy diet score (continuous)

The UE and OB groups were classified as obese, based on either their actBMI or metBMI (Table 1). Although the actBMI was higher in the UE group than in the OB group, the UE and OB groups showed similar risks of mortality, CVD events, and age-related eye diseases after full adjustment (all P > 0.05 in Model 2 of Table 2).

### Subgroup and ROC analyses in UKB

Sex-based subgroup analyses showed that the risks of all-cause mortality, heart failure, and coronary heart disease were more profound among females, while the risks of CVD mortality and myocardial infarction were more profound among males when comparing OE to NW groups (Additional file 1: Table S6). The subgroup analysis of a healthy diet demonstrated that individuals with an unhealthy diet mode (healthy diet score < 4) were more likely to experience obesity-related metabolic risks in all-cause and CVD-cause mortality, myocardial infarction, and coronary heart disease, than those with a healthy diet mode (healthy diet score ≥ 4) (Additional file 1: Table S7). Furthermore, the ROC curves showed that metBMI, combined with WHR and serum creatinine, had considerable performance in distinguishing NW from OE, OB, and UE groups (Additional file 1: Fig. S2).

### Abdominal obesity and metabolic WHR models in UKB

Understanding the heterogeneous nature of obesity requires analysing the distribution of fat in the body. Therefore, we used an alternative indicator (WHR) to assess central obesity in the UKB cohort using the same scheme as the metabolome-based BMI models. Individuals with an overestimated WHR showed an increased risk of developing CVD-cause mortality, myocardial infarction, and coronary heart disease compared to those with a normal metabolic WHR (Additional file 1: Fig. S3 and Tables S8–S9).

### Extrapolating the metabolic signature of obesity in the GDES

In order to validate the utility of obesity metabolic fingerprints for discriminating CVD risk, we used LC/MS assays in the GDES cohort. Of the 592 participants with T2D, 99 (16.7%) experienced a CVD event during the 4-year follow-up. We identified 64 serum metabolites as obesity metabolic fingerprints (Additional file 1: Table S10). Incorporating these metabolites led to a significant improvement in the discriminative performance for CVD stratification over clinical indicators (Additional file 1: Fig. S4).

## Discussion

By leveraging 249 metabolites and 12-year follow-up of 89,830 individuals, five subtypes according to gaps between metBMI and actBMI were obtained in the present study, including two extreme phenotypes (OE, metabolically unhealthy normal weight; UE, metabolically healthy obesity). Abnormally increased metBMI was significantly associated with mortality, CVD, and age-related eye diseases. Regardless of baseline actBMI, obese-related metabolites imposed a higher risk of developing multi-disease outcomes than those with healthy metabolic profiles. The varying risk of CVD across metabolomic-defined obesity was further verified in an independent Chinese population using a different metabolomic profiling method.

The metabolome-predicted BMI achieved good performance, which is consistent with those of previous studies and confirm metabolites’ association with BMI-defined obesity [10, 11, 20]. Unhealthy metabolic profiles have been previously shown to increase the risk of CVDs. In the Framingham Study cohort, participants free of CVD at 50 years with elevated levels of cholesterol, blood pressure, BMI, diabetes, and smoking were at significantly higher risk of lifetime CVD and shorter lifespan. Notably, lifetime CVD risk in females gradually increased across normal weight, overweight, and obesity categories (35.3%, 42.5%, and 43.0%, respectively) [21]. A meta-analysis of 9 cohorts and 3 nested case–control studies showed several metabolites, such as lipids, amino acids, and others, positively or negatively associated with CVD events [22]. Moreover, 19 metabolites, including sugar substitutes and erythritol, were positively or negatively associated with incident coronary heart disease in the Atherosclerosis Risk in Communities (ARIC) study [23]. Our study analyzed a population that was free from CVDs and had a 12-year follow-up duration. We observed that individuals with unhealthy metabolic profiles were more likely to have higher levels of serum lipids, blood pressure, and glucose. Furthermore, we found that an increase in obesity-related metabolites was associated with a higher risk of CVDs, regardless of the individual's actual BMI.

However, our study revealed some inconsistent findings when compared to previous studies. Specifically, our study identified glycoprotein acetyl, albumin, tyrosine, valine, and glutamine as the metabolites with the strongest correlation coefficients. While other studies have shown a high correlation between some amino acid metabolites and CVD risk, they did not find valine or tyrosine to be strongly associated with CVD [22]. It is worth noting that valine and tyrosine are believed to contribute to severe obesity by affecting metabolism, particularly through triglyceride hydrolysis and hepatic mitochondrial dysfunction [24–26]. Several reasons may account for the inconsistent findings between our study and others. For instance, some studies did not exclude individuals with prevalent CVDs at baseline, which may have affected the accuracy of their results, as baseline CVDs are likely to have already influenced the levels of metabolites [22]. Furthermore, different analytical processes used in serum metabolome profiling techniques, such as mass spectrometry (MS) and NMR, may lead to different assessments of metabolites due to technique qualifications [22, 23].

Metabolomics is a promising tool for discovering cardiovascular biomarkers [9]. It enables the identification and quantification of small molecules that reflect the organism's state at a given point in time, as well as the fingerprinting of disease and preclinical disease states [22]. As summarized previously, acylcarnitines and dicarboxylacylcarnitines, trimethylamine *N*-oxide, and various amino acids such as phenylalanine and glutamate, and several lipid classes are associated with CVD risk [22]. Interestingly, several types of metabolites, such as dicarboxylcarnitines and acylcarnitines, plasma branched and aromatic amino acids, phenylalanine, a-hydroxybutyrate, and ceramides, can be used to fingerprint cardiovascular risk factors such as obesity, insulin resistance, and diabetes [22]. Most amino acids were increased in obesity subjects and were confirmed among people with nonalcoholic fatty liver disease (NAFLD) subjects due to increased insulin resistance and protein catabolism [27]. Additionally, an experiment with diet-induced obesity rats supported the anti-inflammatory effect of tyrosine and tryptophan in suppressing the production of proinflammatory cytokines, as well as the effect of tyrosine supplementation on metabolic homeostasis and normalizing triglycerides and LDL cholesterol levels [28]. Importantly, the metabolite risk score could significantly improve model predictive performance in the assessment of 30-year coronary heart disease risk prediction [23].

People with a normal BMI are commonly ignored in major disease prevention guidelines. Although the actBMI in the OE group was lower than the NW group, a significantly higher risk of mortality was noted in the OE group. The NHANES-III finding that metabolically healthy obesity does not increase the risk of CVD mortality [29]. The risks of AMD were significantly higher in the OE group compared with the NW group, indicating that normal-weight and obese individuals have metabolites that put them at risk of accelerated ageing and abnormal metabolism in systemic and local tissues. Regardless of baseline BMI, individuals carrying an obesity-associated metabolomic profile are at greater risk of vascular and ocular diseases.

The UE and OB groups were obese according to actBMI and metBMI, and the actBMI of the UE group was even significantly higher than that of the OB group (36.6 vs. 30.9 kg/m^2^). However, the risk of morbidity was similar between the UE and OB groups. These findings indicated that the absence of obesity metabolites reduced the likelihood of mortality and muti-disease even in individuals with an actual abnormal BMI. Metabolomic profiling can help to population stratification beyond the anthropometrics.

Our study underscores the importance of a healthy and high-quality diet in reducing the risk of cardiometabolic and ocular diseases. A healthy diet is typically characterized by high fiber content, and adherence to a Mediterranean-style healthy diet has been shown to be associated with beneficial changes in gut microbiome composition [30, 31]. Conversely, an unhealthy diet that is often high in fat and sugar has been associated with negative alterations in the microbiota [32]. Therefore, consuming a healthy diet with at least 4 out of 7 commonly eaten food groups on a weekly basis may help to protect against metabolic disturbances and promote cardiometabolic health [19].

The use of metabolic BMI as an indicator for obesity appears to be more accurate than anthropometric BMI. Other anthropometric measurements, such as waist circumference (WC), waist-to-height ratio (WHR), and central obesity, have also been shown to be useful in defining obesity and metabolic disturbances. For example, individuals in the normal actBMI category with a large WC, WHR, and waist-to-height ratio are at higher risk of CVD events and have been suggested as novel indicators for assessing cardiovascular and ocular health, independent of BMI [33–37]. Furthermore, people with both central and general obesity are prone to high urinary albumin-creatinine ratio. Our study found that combining metBMI, WHR, and serum creatinine provided considerable performance in distinguishing metBMI subgroups. Additionally, our results suggested that an alternative indicator for assessing central obesity may be useful in evaluating the obesity-related metabolic risk in the development of CVDs. Further research is needed to validate these findings.

The metabolomics associated with each class suggest that health statuses differ greatly in each BMI group, and metabolomics provides a more accurate representation for quantifying an obese phenotype with likely future problematic health. This study suggested that the metabolomics might partly explained the obesity paradox [5]. Currently, most guidelines pressure obese and overweight individuals to receive lifestyle and medical treatments to improve their metabolic state. For decades, anthropometric BMI has been a convenient method to determine an individual’s health status. However, this practice has stigmatised individuals who fall outside the healthy weight range and can misdirect health practitioners to believe that a normal-weight BMI translates to good health. Considering that obesity-associated metabolites are not significantly influenced by genetic factors and lifestyle interventions can effectively improve metabolism, this study stresses the importance of not judging by size or weight, encouraging instead a metabolomics-based definition of obesity to guide management [10, 11, 38].

A significant strength of this study is the collaborative validation carried out among the Chinese population, enabling the generalizability of the findings. Furthermore, future studies involving additional ethnicities are warranted to further validate and augment these findings. This study has some limitations. First, because metabolomic information was only available at baseline, the correlations between dynamic metBMIs and endpoints were not assessed. Despite this, a single glimpse at the metabolome, even 12 years earlier, provided strong causal links to the outcomes investigated. Second, the UKB used high-throughput NMR techniques that limited the breadth of obesity-related metabolites included for analysis. Other techniques not used by this study, such as MS, chromatography, and electrophoresis, may yield other metabolites for a deeper investigation. Third, the classification criterion for the abnormal group was arbitrarily defined by a prediction error of 5 kg/m^2^ for metBMI, which was based on and referenced from previous studies [10, 11]. The actual optimal cut-off criterion remains to be explored by further studies.

## Conclusions

In summary, blood metabolomics can provide insight into the phenotyping of obesity defined by BMI and reveal inter-population variability. Individuals carrying obesity-related metabolites have a higher risk of developing various diseases compared with healthy metabolomes. By leveraging the gaps between metBMI and actBMI, ‘healthily obese’ and ‘unhealthily lean’ phenotypes were validated in this large cohort study. Conducting follow-up studies of blood metabolomics can reflect the health implications of altered obesity heterogeneity in populations. This highlights the significance of obesity-related metabolic fingerprints for predictive and preventive medicine.

## Supplementary Information

**Additional file 1. **Additional Figures, Tables and methods.

## Data Availability

Data and materials are available via UK Biobank at http://www.ukbiobank.ac.uk/.

## Abbreviations

DR: Diabetic retinopathy
CVD: Cardiovascular disease
UKB: UK Biobank
HbA1c: Hemoglobin A1c
BMI: Body mass index
SD: Standard deviation
HR: Hazard ratio
CI: Confidence interval
HDL: High-density lipoprotein
IDL: Intermediate-density lipoprotein
VLDL: Very low-density lipoprotein
NW: Normal weight of metabolic BMI
OW: Overweight of metabolic BMI
OB: Obesity of metabolic BMI
OE: Metabolome-defined BMI over than actual BMI
UE: Metabolome-defined BMI under than actual BMI
AMD: Age-related macular degeneration
