# Supplementary material for: Metabolomic phenotyping of obesity for profiling cardiovascular and ocular diseases
Source: J Transl Med. 2023 Jun 12;21:384. doi: 10.1186/s12967-023-04244-x (PMC10262415; doi:10.1186/s12967-023-04244-x)
Supplement: Supplementary file 1 — Additional file 1. Additional Figures, Tables and methods. [file 12967_2023_4244_MOESM1_ESM.docx]

**Supplementary material**

**Supplementary methods: 4**

**Supplementary figures: 4**

**Supplementary tables: 10**

**Contents**

[SUPPLEMENTARY FIGURES 2](#_Toc134379232)

[Figure S1. Survival plots showing the risks of cardiovascular and ocular diseases outcomes comparing OB versus UE groups. 2](#_Toc134379233)

[Figure S2. ROC plots for distinguishing metabolic BMI subgroups in application dataset. 3](#_Toc134379234)

[Figure S3. Distribution of metabolomic WHR, actual WHR, and metabolites used to predict WHR in the applicative set. 4](#_Toc134379235)

[Figure S4. Receiver operator characteristic (ROC) curves for obesity-related metabolites in predicting 4-year incident cardiovascular events. 5](#_Toc134379236)

[SUPPLEMENTARY TABLES 6](#_Toc134379237)

[Table S1. List of NMR-based metabolomic biomarkers in the UK Biobank. 6](#_Toc134379238)

[Table S2. List and formulae for calculating ratios of metabolomic measurements in the UK Biobank. 14](#_Toc134379239)

[Table S3. ICD and OPCS codes for identifying incidents vascular and ocular outcomes. 17](#_Toc134379240)

[Table S4. Baseline characteristics of included participants by dataset. 18](#_Toc134379241)

[Table S5. Incident events and number in the application set. 19](#_Toc134379242)

[Table S6. Subgroup analysis by sex of associations between overestimated metabolic BMI and incident endpoints in application set. 20](#_Toc134379243)

[Table S7. Subgroup analysis by diet score of associations between overestimated metabolic BMI and incident endpoints in application set. 21](#_Toc134379244)

[Table S8. Baseline characteristics of the included participants in the WHR model applicative set. 22](#_Toc134379245)

[Table S9. Associations of metabolome WHR phenotypes with risks of cardiovascular and ocular disease outcomes in the applicative set. 23](#_Toc134379246)

[Table S10. List summarizing metabolic obesity fingerprints identified in the GDES cohort using LC/MS profiling. 24](#_Toc134379247)

[SUPPLEMENTARY Method 27](#_Toc134379248)

[Method S1. Assessment of diet. 27](#_Toc134379249)

[Method S2. Profiling details and quality control processes of 1H-NMR profiling in UKB. 28](#_Toc134379250)

[Method S3. Profiling details and quality control processes of LC/MS profiling in the GDES cohort. 29](#_Toc134379251)

[Method S4. Metabolome-based BMI and WHR models. 30](#_Toc134379252)

#

# SUPPLEMENTARY FIGURES

## Figure S1. Survival plots showing the risks of cardiovascular and ocular diseases outcomes comparing OB versus UE groups.


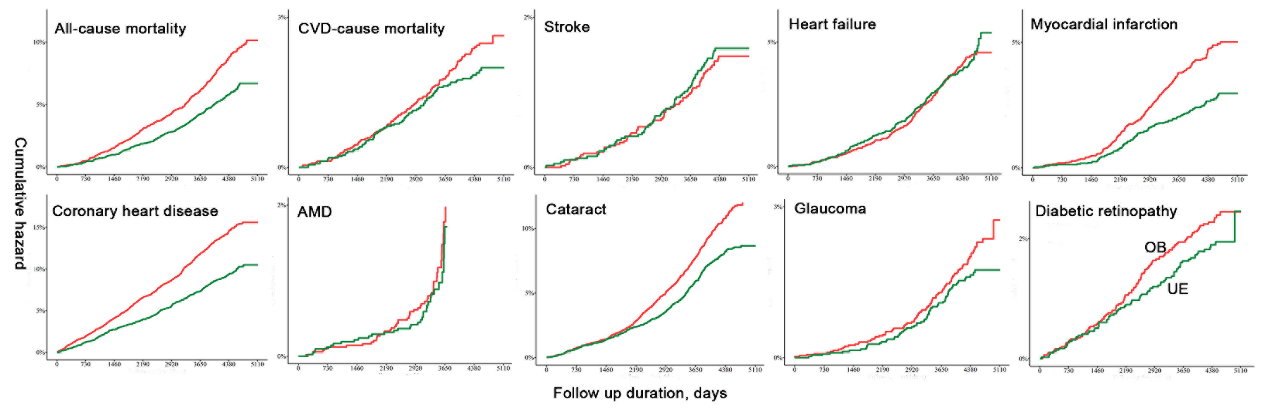


OB= obesity of metabolic BMI; UE= metabolome-defined BMI under than actual BMI; CVD= cardiovascular disease; AMD= age-related macular degeneration.

The log-rank tests for NW and OE group show P-values for the outcomes: all-cause mortality: <0.001; CVD mortality: 0.105; stroke: 0.654; heart failure: 0.910; myocardial infarction: <0.001; coronary heart disease: <0.001; AMD: 0.442; cataract: <0.001; glaucoma: 0.117; DR: 0.176.

## Figure S2. ROC plots for distinguishing metabolic BMI subgroups in application dataset.

**

**

ROC= receiver operating characteristic; AUC= area under the curve; BMI= body mass index; NW= normal weight of metabolic BMI, OW= overweight of metabolic BMI; OB= obesity of metabolic BMI; OE= metabolome-defined BMI over than actual BMI; UE= metabolome-defined BMI under than actual BMI.

ROC curves were created using factors of metabolic BMI, waist-to-hip ratio, and serum creatinine. of the model distinguish NW from OE, OB, and UE groups, with AUC of 0.895 (0.889, 0.902), 0.870 (0.862, 0.877), and 0.858 (0.850, 0.866), respectively.

## Figure S3. Distribution of metabolomic WHR, actual WHR, and metabolites used to predict WHR in the applicative set.





(A) subgrouping for all individuals in application dataset, the correlation r^2^ value is 0.477; (B) distribution of actWHR across groups; (C) refers to the top 30 metabolites with high correlation ratio selected by ridge regression model. WHR=waist-to-hip ratio; NW_pre= normal of metabolic WHR, OB_pre= obesity of metabolic WHR; OE_pre= metabolome-defined WHR over than actual WHR; UE_pre= metabolome-defined WHR under than actual WHR.

## Figure S4. Receiver operator characteristic (ROC) curves for obesity-related metabolites in predicting 4-year incident cardiovascular events.


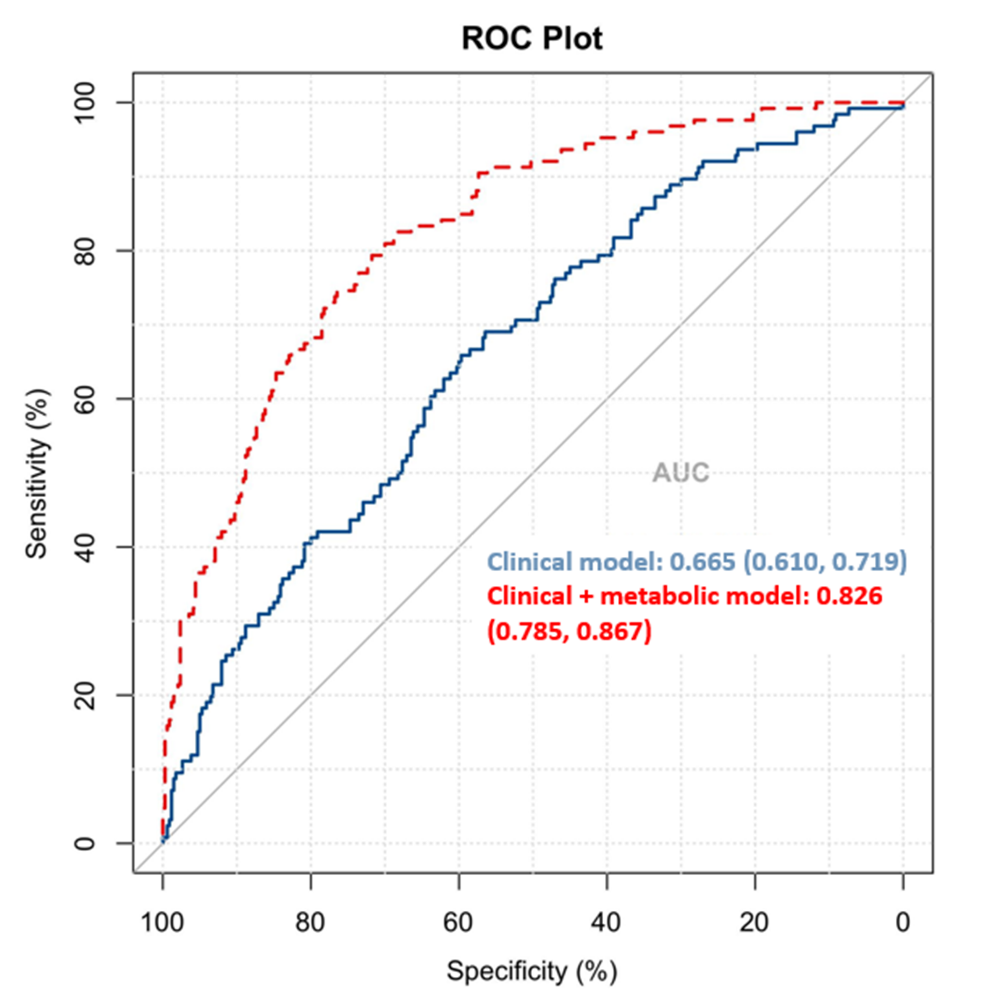


P-value for comparison: <0.001

Traditional risk factors: age, sex, total cholesterol, high-density lipoprotein, current smoker and alcohol-drinker, systolic blood pressure.

Obesity-related metabolites for incident 4-year cardiovascular diseases: L-Arginine, L-Aspartic Acid, L-Citrulline, L-Glutamic Acid, L-Serine, L-Tryptophan, 3-Hydroxy-3-Methylpentane-1,5-Dioic Acid, 3-N-Methyl-L-Histidine, Glutathione Oxidized, Hexanoyl Glycine, L-Asparagine Anhydrous, L-Cysteine, L-Homocitrulline, L-Saccharopine, N-Acetyl-L-Leucine, N-Acetyl-L-Tyrosine, N-Isovaleroylglycine, N-Propionylglycine, O-Phospho-L-Serine, Phenylacetyl-L-Glutamine, S-(5-Adenosy)-L-Homocysteine, γ-Glu-Cys, 3-(4-hydroxyphenyl)acrylaldehyde, Benzoic Acid, Terephthalic Acid, Phthalic Acid, 2-Picolinic Acid, 4-Pyridoxic Acid, 6-Hydroxynicotinic Acid, Glycolithocholic acid, Hyodeoxycholic acid, Glycocholic Acid, Glycochenodeoxycholic Acid, Chenodeoxycholic Acid, 4-Hydroxy-3-methoxybenzaldehyde, 1-Methylhistidine, Xanthine, 5-Methylcytosine, Adenosine 5'-Monophosphate, Hypoxanthine, Uridine, Β-Nicotinamide Mononucleotide, Epinephrine, Pyrroloquinoline Quinone, Cis-Aconitic Acid, Melatonin, Tryptamine, Dl-Threitol, D-Sorbitol, D-Glucose, D-Erythronolactone, L-Fucose, L-Rhamnose, Maltose, N-Acetyl-D-Glucosamine, D-Glyceric Acid, L-Gulonolactone, Orotic Acid, Pantothenate, 1,7-Dimethyluric Acid, 2,6-Diaminopimelic acid, Taurine, 2-Hydroxybutanoic Acid, 3-Hydroxy-3-Methyl Butyric Acid.

# SUPPLEMENTARY TABLES

## Table S1. List of NMR-based metabolomic biomarkers in the UK Biobank.

| **Field ID** | **Title** | **Units** | **Group** | **Subgroup** |
| --- | --- | --- | --- | --- |
| 23400 | Total Cholesterol | mmol/L | Cholesterol | NA |
| 23401 | Total Cholesterol Minus HDL-C | mmol/L | Cholesterol | NA |
| 23402 | Remnant Cholesterol (Non-HDL, Non-LDL -Cholesterol) | mmol/L | Cholesterol | NA |
| 23403 | VLDL Cholesterol | mmol/L | Cholesterol | NA |
| 23404 | Clinical LDL Cholesterol | mmol/L | Cholesterol | NA |
| 23405 | LDL Cholesterol | mmol/L | Cholesterol | NA |
| 23406 | HDL Cholesterol | mmol/L | Cholesterol | NA |
| 23407 | Total Triglycerides | mmol/L | Triglycerides | NA |
| 23408 | Triglycerides in VLDL | mmol/L | Triglycerides | NA |
| 23409 | Triglycerides in LDL | mmol/L | Triglycerides | NA |
| 23410 | Triglycerides in HDL | mmol/L | Triglycerides | NA |
| 23411 | Total Phospholipids in Lipoprotein Particles | mmol/L | Phospholipids | NA |
| 23412 | Phospholipids in VLDL | mmol/L | Phospholipids | NA |
| 23413 | Phospholipids in LDL | mmol/L | Phospholipids | NA |
| 23414 | Phospholipids in HDL | mmol/L | Phospholipids | NA |
| 23415 | Total Esterified Cholesterol | mmol/L | Cholesteryl esters | NA |
| 23416 | Cholesteryl Esters in VLDL | mmol/L | Cholesteryl esters | NA |
| 23417 | Cholesteryl Esters in LDL | mmol/L | Cholesteryl esters | NA |
| 23418 | Cholesteryl Esters in HDL | mmol/L | Cholesteryl esters | NA |
| 23419 | Total Free Cholesterol | mmol/L | Free cholesterol | NA |
| 23420 | Free Cholesterol in VLDL | mmol/L | Free cholesterol | NA |
| 23421 | Free Cholesterol in LDL | mmol/L | Free cholesterol | NA |
| 23422 | Free Cholesterol in HDL | mmol/L | Free cholesterol | NA |
| 23423 | Total Lipids in Lipoprotein Particles | mmol/L | Total lipids | NA |
| 23424 | Total Lipids in VLDL | mmol/L | Total lipids | NA |
| 23425 | Total Lipids in LDL | mmol/L | Total lipids | NA |
| 23426 | Total Lipids in HDL | mmol/L | Total lipids | NA |
| 23427 | Total Concentration of Lipoprotein Particles | mmol/L | Lipoprotein particle concentrations | NA |
| 23428 | Concentration of VLDL Particles | mmol/L | Lipoprotein particle concentrations | NA |
| 23429 | Concentration of LDL Particles | mmol/L | Lipoprotein particle concentrations | NA |
| 23430 | Concentration of HDL Particles | mmol/L | Lipoprotein particle concentrations | NA |
| 23431 | Average Diameter for VLDL Particles | nm | Lipoprotein particle sizes | NA |
| 23432 | Average Diameter for LDL Particles | nm | Lipoprotein particle sizes | NA |
| 23433 | Average Diameter for HDL Particles | nm | Lipoprotein particle sizes | NA |
| 23434 | Phosphoglycerides | mmol/L | Other lipids | NA |
| 23436 | Total Cholines | mmol/L | Other lipids | NA |
| 23437 | Phosphatidylcholines | mmol/L | Other lipids | NA |
| 23438 | Sphingomyelins | mmol/L | Other lipids | NA |
| 23439 | Apolipoprotein B | g/l | Apolipoproteins | NA |
| 23440 | Apolipoprotein A1 | g/l | Apolipoproteins | NA |
| 23442 | Total Fatty Acids | mmol/L | Fatty acids | NA |
| 23443 | Degree of Unsaturation | degree | Fatty acids | NA |
| 23444 | Omega-3 Fatty Acids | mmol/L | Fatty acids | NA |
| 23445 | Omega-6 Fatty Acids | mmol/L | Fatty acids | NA |
| 23446 | Polyunsaturated Fatty Acids | mmol/L | Fatty acids | NA |
| 23447 | Monounsaturated Fatty Acids | mmol/L | Fatty acids | NA |
| 23448 | Saturated Fatty Acids | mmol/L | Fatty acids | NA |
| 23449 | Linoleic Acid | mmol/L | Fatty acids | NA |
| 23450 | Docosahexaenoic Acid | mmol/L | Fatty acids | NA |
| 23460 | Alanine | mmol/L | Amino acids | NA |
| 23461 | Glutamine | mmol/L | Amino acids | NA |
| 23462 | Glycine | mmol/L | Amino acids | NA |
| 23463 | Histidine | mmol/L | Amino acids | NA |
| 23464 | Total Concentration of Branched-Chain Amino Acids (Leucine + Isoleucine + Valine) | mmol/L | Amino acids | Branched-chain amino acids |
| 23465 | Isoleucine | mmol/L | Amino acids | Branched-chain amino acids |
| 23466 | Leucine | mmol/L | Amino acids | Branched-chain amino acids |
| 23467 | Valine | mmol/L | Amino acids | Branched-chain amino acids |
| 23468 | Phenylalanine | mmol/L | Amino acids | Aromatic amino acids |
| 23469 | Tyrosine | mmol/L | Amino acids | Aromatic amino acids |
| 23470 | Glucose | mmol/L | Glycolysis related metabolites | NA |
| 23471 | Lactate | mmol/L | Glycolysis related metabolites | NA |
| 23472 | Pyruvate | mmol/L | Glycolysis related metabolites | NA |
| 23473 | Citrate | mmol/L | Glycolysis related metabolites | NA |
| 23474 | 3-Hydroxybutyrate | mmol/L | Ketone bodies | NA |
| 23475 | Acetate | mmol/L | Ketone bodies | NA |
| 23476 | Acetoacetate | mmol/L | Ketone bodies | NA |
| 23477 | Acetone | mmol/L | Ketone bodies | NA |
| 23478 | Creatinine | mmol/L | Fluid balance | NA |
| 23479 | Albumin | g/l | Fluid balance | NA |
| 23480 | Glycoprotein Acetyls | mmol/L | Inflammation | NA |
| 23481 | Concentration of Chylomicrons and Extremely Large VLDL Particles | mmol/L | Lipoprotein subclasses | Chylomicrons and extremely large VLDL (particle diameters from 75 nm upwards) |
| 23482 | Total Lipids in Chylomicrons and Extremely Large VLDL | mmol/L | Lipoprotein subclasses | Chylomicrons and extremely large VLDL (particle diameters from 75 nm upwards) |
| 23483 | Phospholipids in Chylomicrons and Extremely Large VLDL | mmol/L | Lipoprotein subclasses | Chylomicrons and extremely large VLDL (particle diameters from 75 nm upwards) |
| 23484 | Cholesterol in Chylomicrons and Extremely Large VLDL | mmol/L | Lipoprotein subclasses | Chylomicrons and extremely large VLDL (particle diameters from 75 nm upwards) |
| 23485 | Cholesteryl Esters in Chylomicrons and Extremely Large VLDL | mmol/L | Lipoprotein subclasses | Chylomicrons and extremely large VLDL (particle diameters from 75 nm upwards) |
| 23486 | Free Cholesterol in Chylomicrons and Extremely Large VLDL | mmol/L | Lipoprotein subclasses | Chylomicrons and extremely large VLDL (particle diameters from 75 nm upwards) |
| 23487 | Triglycerides in Chylomicrons and Extremely Large VLDL | mmol/L | Lipoprotein subclasses | Chylomicrons and extremely large VLDL (particle diameters from 75 nm upwards) |
| 23488 | Concentration of Very Large VLDL Particles | mmol/L | Lipoprotein subclasses | Very large VLDL (average diameter 64 nm) |
| 23489 | Total Lipids in Very Large VLDL | mmol/L | Lipoprotein subclasses | Very large VLDL (average diameter 64 nm) |
| 23490 | Phospholipids in Very Large VLDL | mmol/L | Lipoprotein subclasses | Very large VLDL (average diameter 64 nm) |
| 23491 | Cholesterol in Very Large VLDL | mmol/L | Lipoprotein subclasses | Very large VLDL (average diameter 64 nm) |
| 23492 | Cholesteryl Esters in Very Large VLDL | mmol/L | Lipoprotein subclasses | Very large VLDL (average diameter 64 nm) |
| 23493 | Free Cholesterol in Very Large VLDL | mmol/L | Lipoprotein subclasses | Very large VLDL (average diameter 64 nm) |
| 23494 | Triglycerides in Very Large VLDL | mmol/L | Lipoprotein subclasses | Very large VLDL (average diameter 64 nm) |
| 23495 | Concentration of Large VLDL Particles | mmol/L | Lipoprotein subclasses | Large VLDL (average diameter 53.6 nm) |
| 23496 | Total Lipids in Large VLDL | mmol/L | Lipoprotein subclasses | Large VLDL (average diameter 53.6 nm) |
| 23497 | Phospholipids in Large VLDL | mmol/L | Lipoprotein subclasses | Large VLDL (average diameter 53.6 nm) |
| 23498 | Cholesterol in Large VLDL | mmol/L | Lipoprotein subclasses | Large VLDL (average diameter 53.6 nm) |
| 23499 | Cholesteryl Esters in Large VLDL | mmol/L | Lipoprotein subclasses | Large VLDL (average diameter 53.6 nm) |
| 23500 | Free Cholesterol in Large VLDL | mmol/L | Lipoprotein subclasses | Large VLDL (average diameter 53.6 nm) |
| 23501 | Triglycerides in Large VLDL | mmol/L | Lipoprotein subclasses | Large VLDL (average diameter 53.6 nm) |
| 23502 | Concentration of Medium VLDL Particles | mmol/L | Lipoprotein subclasses | Medium VLDL (average diameter 44.5 nm) |
| 23503 | Total Lipids in Medium VLDL | mmol/L | Lipoprotein subclasses | Medium VLDL (average diameter 44.5 nm) |
| 23504 | Phospholipids in Medium VLDL | mmol/L | Lipoprotein subclasses | Medium VLDL (average diameter 44.5 nm) |
| 23505 | Cholesterol in Medium VLDL | mmol/L | Lipoprotein subclasses | Medium VLDL (average diameter 44.5 nm) |
| 23506 | Cholesteryl Esters in Medium VLDL | mmol/L | Lipoprotein subclasses | Medium VLDL (average diameter 44.5 nm) |
| 23507 | Free Cholesterol in Medium VLDL | mmol/L | Lipoprotein subclasses | Medium VLDL (average diameter 44.5 nm) |
| 23508 | Triglycerides in Medium VLDL | mmol/L | Lipoprotein subclasses | Medium VLDL (average diameter 44.5 nm) |
| 23509 | Concentration of Small VLDL Particles | mmol/L | Lipoprotein subclasses | Small VLDL (average diameter 36.8 nm) |
| 23510 | Total Lipids in Small VLDL | mmol/L | Lipoprotein subclasses | Small VLDL (average diameter 36.8 nm) |
| 23511 | Phospholipids in Small VLDL | mmol/L | Lipoprotein subclasses | Small VLDL (average diameter 36.8 nm) |
| 23512 | Cholesterol in Small VLDL | mmol/L | Lipoprotein subclasses | Small VLDL (average diameter 36.8 nm) |
| 23513 | Cholesteryl Esters in Small VLDL | mmol/L | Lipoprotein subclasses | Small VLDL (average diameter 36.8 nm) |
| 23514 | Free Cholesterol in Small VLDL | mmol/L | Lipoprotein subclasses | Small VLDL (average diameter 36.8 nm) |
| 23515 | Triglycerides in Small VLDL | mmol/L | Lipoprotein subclasses | Small VLDL (average diameter 36.8 nm) |
| 23516 | Concentration of Very Small VLDL Particles | mmol/L | Lipoprotein subclasses | Very small VLDL (average diameter 31.3 nm) |
| 23517 | Total Lipids in Very Small VLDL | mmol/L | Lipoprotein subclasses | Very small VLDL (average diameter 31.3 nm) |
| 23518 | Phospholipids in Very Small VLDL | mmol/L | Lipoprotein subclasses | Very small VLDL (average diameter 31.3 nm) |
| 23519 | Cholesterol in Very Small VLDL | mmol/L | Lipoprotein subclasses | Very small VLDL (average diameter 31.3 nm) |
| 23520 | Cholesteryl Esters in Very Small VLDL | mmol/L | Lipoprotein subclasses | Very small VLDL (average diameter 31.3 nm) |
| 23521 | Free Cholesterol in Very Small VLDL | mmol/L | Lipoprotein subclasses | Very small VLDL (average diameter 31.3 nm) |
| 23522 | Triglycerides in Very Small VLDL | mmol/L | Lipoprotein subclasses | Very small VLDL (average diameter 31.3 nm) |
| 23523 | Concentration of IDL Particles | mmol/L | Lipoprotein subclasses | IDL (average diameter 28.6 nm) |
| 23524 | Total Lipids in IDL | mmol/L | Lipoprotein subclasses | IDL (average diameter 28.6 nm) |
| 23525 | Phospholipids in IDL | mmol/L | Lipoprotein subclasses | IDL (average diameter 28.6 nm) |
| 23526 | Cholesterol in IDL | mmol/L | Lipoprotein subclasses | IDL (average diameter 28.6 nm) |
| 23527 | Cholesteryl Esters in IDL | mmol/L | Lipoprotein subclasses | IDL (average diameter 28.6 nm) |
| 23528 | Free Cholesterol in IDL | mmol/L | Lipoprotein subclasses | IDL (average diameter 28.6 nm) |
| 23529 | Triglycerides in IDL | mmol/L | Lipoprotein subclasses | IDL (average diameter 28.6 nm) |
| 23530 | Concentration of Large LDL Particles | mmol/L | Lipoprotein subclasses | Large LDL (average diameter 25.5 nm) |
| 23531 | Total Lipids in Large LDL | mmol/L | Lipoprotein subclasses | Large LDL (average diameter 25.5 nm) |
| 23532 | Phospholipids in Large LDL | mmol/L | Lipoprotein subclasses | Large LDL (average diameter 25.5 nm) |
| 23533 | Cholesterol in Large LDL | mmol/L | Lipoprotein subclasses | Large LDL (average diameter 25.5 nm) |
| 23534 | Cholesteryl Esters in Large LDL | mmol/L | Lipoprotein subclasses | Large LDL (average diameter 25.5 nm) |
| 23535 | Free Cholesterol in Large LDL | mmol/L | Lipoprotein subclasses | Large LDL (average diameter 25.5 nm) |
| 23536 | Triglycerides in Large LDL | mmol/L | Lipoprotein subclasses | Large LDL (average diameter 25.5 nm) |
| 23537 | Concentration of Medium LDL Particles | mmol/L | Lipoprotein subclasses | Medium LDL (average diameter 23 nm) |
| 23538 | Total Lipids in Medium LDL | mmol/L | Lipoprotein subclasses | Medium LDL (average diameter 23 nm) |
| 23539 | Phospholipids in Medium LDL | mmol/L | Lipoprotein subclasses | Medium LDL (average diameter 23 nm) |
| 23540 | Cholesterol in Medium LDL | mmol/L | Lipoprotein subclasses | Medium LDL (average diameter 23 nm) |
| 23541 | Cholesteryl Esters in Medium LDL | mmol/L | Lipoprotein subclasses | Medium LDL (average diameter 23 nm) |
| 23542 | Free Cholesterol in Medium LDL | mmol/L | Lipoprotein subclasses | Medium LDL (average diameter 23 nm) |
| 23543 | Triglycerides in Medium LDL | mmol/L | Lipoprotein subclasses | Medium LDL (average diameter 23 nm) |
| 23544 | Concentration of Small LDL Particles | mmol/L | Lipoprotein subclasses | Small LDL (average diameter 18.7 nm) |
| 23545 | Total Lipids in Small LDL | mmol/L | Lipoprotein subclasses | Small LDL (average diameter 18.7 nm) |
| 23546 | Phospholipids in Small LDL | mmol/L | Lipoprotein subclasses | Small LDL (average diameter 18.7 nm) |
| 23547 | Cholesterol in Small LDL | mmol/L | Lipoprotein subclasses | Small LDL (average diameter 18.7 nm) |
| 23548 | Cholesteryl Esters in Small LDL | mmol/L | Lipoprotein subclasses | Small LDL (average diameter 18.7 nm) |
| 23549 | Free Cholesterol in Small LDL | mmol/L | Lipoprotein subclasses | Small LDL (average diameter 18.7 nm) |
| 23550 | Triglycerides in Small LDL | mmol/L | Lipoprotein subclasses | Small LDL (average diameter 18.7 nm) |
| 23551 | Concentration of Very Large HDL Particles | mmol/L | Lipoprotein subclasses | Very large HDL (average diameter 14.3 nm) |
| 23552 | Total Lipids in Very Large HDL | mmol/L | Lipoprotein subclasses | Very large HDL (average diameter 14.3 nm) |
| 23553 | Phospholipids in Very Large HDL | mmol/L | Lipoprotein subclasses | Very large HDL (average diameter 14.3 nm) |
| 23554 | Cholesterol in Very Large HDL | mmol/L | Lipoprotein subclasses | Very large HDL (average diameter 14.3 nm) |
| 23555 | Cholesteryl Esters in Very Large HDL | mmol/L | Lipoprotein subclasses | Very large HDL (average diameter 14.3 nm) |
| 23556 | Free Cholesterol in Very Large HDL | mmol/L | Lipoprotein subclasses | Very large HDL (average diameter 14.3 nm) |
| 23557 | Triglycerides in Very Large HDL | mmol/L | Lipoprotein subclasses | Very large HDL (average diameter 14.3 nm) |
| 23558 | Concentration of Large HDL Particles | mmol/L | Lipoprotein subclasses | Large HDL (average diameter 12.1 nm) |
| 23559 | Total Lipids in Large HDL | mmol/L | Lipoprotein subclasses | Large HDL (average diameter 12.1 nm) |
| 23560 | Phospholipids in Large HDL | mmol/L | Lipoprotein subclasses | Large HDL (average diameter 12.1 nm) |
| 23561 | Cholesterol in Large HDL | mmol/L | Lipoprotein subclasses | Large HDL (average diameter 12.1 nm) |
| 23562 | Cholesteryl Esters in Large HDL | mmol/L | Lipoprotein subclasses | Large HDL (average diameter 12.1 nm) |
| 23563 | Free Cholesterol in Large HDL | mmol/L | Lipoprotein subclasses | Large HDL (average diameter 12.1 nm) |
| 23564 | Triglycerides in Large HDL | mmol/L | Lipoprotein subclasses | Large HDL (average diameter 12.1 nm) |
| 23565 | Concentration of Medium HDL Particles | mmol/L | Lipoprotein subclasses | Medium HDL (average diameter 10.9 nm) |
| 23566 | Total Lipids in Medium HDL | mmol/L | Lipoprotein subclasses | Medium HDL (average diameter 10.9 nm) |
| 23567 | Phospholipids in Medium HDL | mmol/L | Lipoprotein subclasses | Medium HDL (average diameter 10.9 nm) |
| 23568 | Cholesterol in Medium HDL | mmol/L | Lipoprotein subclasses | Medium HDL (average diameter 10.9 nm) |
| 23569 | Cholesteryl Esters in Medium HDL | mmol/L | Lipoprotein subclasses | Medium HDL (average diameter 10.9 nm) |
| 23570 | Free Cholesterol in Medium HDL | mmol/L | Lipoprotein subclasses | Medium HDL (average diameter 10.9 nm) |
| 23571 | Triglycerides in Medium HDL | mmol/L | Lipoprotein subclasses | Medium HDL (average diameter 10.9 nm) |
| 23572 | Concentration of Small HDL Particles | mmol/L | Lipoprotein subclasses | Small HDL (average diameter 8.7 nm) |
| 23573 | Total Lipids in Small HDL | mmol/L | Lipoprotein subclasses | Small HDL (average diameter 8.7 nm) |
| 23574 | Phospholipids in Small HDL | mmol/L | Lipoprotein subclasses | Small HDL (average diameter 8.7 nm) |
| 23575 | Cholesterol in Small HDL | mmol/L | Lipoprotein subclasses | Small HDL (average diameter 8.7 nm) |
| 23576 | Cholesteryl Esters in Small HDL | mmol/L | Lipoprotein subclasses | Small HDL (average diameter 8.7 nm) |
| 23577 | Free Cholesterol in Small HDL | mmol/L | Lipoprotein subclasses | Small HDL (average diameter 8.7 nm) |
| 23578 | Triglycerides in Small HDL | mmol/L | Lipoprotein subclasses | Small HDL (average diameter 8.7 nm) |

## Table S2. List and formulae for calculating ratios of metabolomic measurements in the UK Biobank.

| **Biomarker name used in the UK Biobank** | **Unit** | **Field ID** | |
| --- | --- | --- | --- |
|  |  | **Numerator** | **Denominator** |
| Ratio of triglycerides to phosphoglycerides | ratio | 23407 | 23434 |
| Ratio of apolipoprotein B to apolipoprotein A1 | ratio | 23439 | 23440 |
| Ratio of omega-3 fatty acids to total fatty acids | % | 23444 | 23442 |
| Ratio of omega-6 fatty acids to total fatty acids | % | 23445 | 23442 |
| Ratio of polyunsaturated fatty acids to total fatty acids | % | 23446 | 23442 |
| Ratio of monounsaturated fatty acids to total fatty acids | % | 23447 | 23442 |
| Ratio of saturated fatty acids to total fatty acids | % | 23448 | 23442 |
| Ratio of linoleic acid to total fatty acids | % | 23449 | 23442 |
| Ratio of docosahexaenoic acid to total fatty acids | % | 23450 | 23442 |
| Ratio of polyunsaturated fatty acids to monounsaturated fatty acids | ratio | 23446 | 23447 |
| Ratio of omega-6 fatty acids to omega-3 fatty acids | ratio | 23445 | 23444 |
| Phospholipids to total lipids ratio in chylomicrons and Extremely Large VLDL | % | 23483 | 23482 |
| Cholesterol to total lipids ratio in chylomicrons and Extremely Large VLDL | % | 23484 | 23482 |
| Cholesteryl esters to total lipids ratio in chylomicrons and Extremely Large VLDL | % | 23485 | 23482 |
| Free cholesterol to total lipids ratio in chylomicrons and Extremely Large VLDL | % | 23486 | 23482 |
| Triglycerides to total lipids ratio in chylomicrons and Extremely Large VLDL | % | 23487 | 23482 |
| Phospholipids to total lipids ratio in Very Large VLDL | % | 23490 | 23489 |
| Cholesterol to total lipids ratio in Very Large VLDL | % | 23491 | 23489 |
| Cholesteryl esters to total lipids ratio in Very Large VLDL | % | 23492 | 23489 |
| Free cholesterol to total lipids ratio in Very Large VLDL | % | 23493 | 23489 |
| Triglycerides to total lipids ratio in Very Large VLDL | % | 23494 | 23489 |
| Phospholipids to total lipids ratio in Large VLDL | % | 23497 | 23496 |
| Cholesterol to total lipids ratio in Large VLDL | % | 23498 | 23496 |
| Cholesteryl esters to total lipids ratio in Large VLDL | % | 23499 | 23496 |
| Free cholesterol to total lipids ratio in Large VLDL | % | 23500 | 23496 |
| Triglycerides to total lipids ratio in Large VLDL | % | 23501 | 23496 |
| Phospholipids to total lipids ratio in Medium VLDL | % | 23504 | 23503 |
| Cholesterol to total lipids ratio in Medium VLDL | % | 23505 | 23503 |
| Cholesteryl esters to total lipids ratio in Medium VLDL | % | 23506 | 23503 |
| Free cholesterol to total lipids ratio in Medium VLDL | % | 23507 | 23503 |
| Triglycerides to total lipids ratio in Medium VLDL | % | 23508 | 23503 |
| Phospholipids to total lipids ratio in Small VLDL | % | 23511 | 23510 |
| Cholesterol to total lipids ratio in Small VLDL | % | 23512 | 23510 |
| Cholesteryl esters to total lipids ratio in Small VLDL | % | 23513 | 23510 |
| Free cholesterol to total lipids ratio in Small VLDL | % | 23514 | 23510 |
| Triglycerides to total lipids ratio in Small VLDL | % | 23515 | 23510 |
| Phospholipids to total lipids ratio in Very Small VLDL | % | 23518 | 23517 |
| Cholesterol to total lipids ratio in Very Small VLDL | % | 23519 | 23517 |
| Cholesteryl esters to total lipids ratio in Very Small VLDL | % | 23520 | 23517 |
| Free cholesterol to total lipids ratio in Very Small VLDL | % | 23521 | 23517 |
| Triglycerides to total lipids ratio in Very Small VLDL | % | 23522 | 23517 |
| Phospholipids to total lipids ratio in IDL | % | 23525 | 23524 |
| Cholesterol to total lipids ratio in IDL | % | 23526 | 23524 |
| Cholesteryl esters to total lipids ratio in IDL | % | 23527 | 23524 |
| Free cholesterol to total lipids ratio in IDL | % | 23528 | 23524 |
| Triglycerides to total lipids ratio in IDL | % | 23529 | 23524 |
| Phospholipids to total lipids ratio in Large LDL | % | 23532 | 23531 |
| Cholesterol to total lipids ratio in Large LDL | % | 23533 | 23531 |
| Cholesteryl esters to total lipids ratio in Large LDL | % | 23534 | 23531 |
| Free cholesterol to total lipids ratio in Large LDL | % | 23535 | 23531 |
| Triglycerides to total lipids ratio in Large LDL | % | 23536 | 23531 |
| Phospholipids to total lipids ratio in Medium LDL | % | 23539 | 23538 |
| Cholesterol to total lipids ratio in Medium LDL | % | 23540 | 23538 |
| Cholesteryl esters to total lipids ratio in Medium LDL | % | 23541 | 23538 |
| Free cholesterol to total lipids ratio in Medium LDL | % | 23542 | 23538 |
| Triglycerides to total lipids ratio in Medium LDL | % | 23543 | 23538 |
| Phospholipids to total lipids ratio in Small LDL | % | 23546 | 23545 |
| Cholesterol to total lipids ratio in Small LDL | % | 23547 | 23545 |
| Cholesteryl esters to total lipids ratio in Small LDL | % | 23548 | 23545 |
| Free cholesterol to total lipids ratio in Small LDL | % | 23549 | 23545 |
| Triglycerides to total lipids ratio in Small LDL | % | 23550 | 23545 |
| Phospholipids to total lipids ratio in Very Large HDL | % | 23553 | 23552 |
| Cholesterol to total lipids ratio in Very Large HDL | % | 23554 | 23552 |
| Cholesteryl esters to total lipids ratio in Very Large HDL | % | 23555 | 23552 |
| Free cholesterol to total lipids ratio in Very Large HDL | % | 23556 | 23552 |
| Triglycerides to total lipids ratio in Very Large HDL | % | 23557 | 23552 |
| Phospholipids to total lipids ratio in Large HDL | % | 23560 | 23559 |
| Cholesterol to total lipids ratio in Large HDL | % | 23561 | 23559 |
| Cholesteryl esters to total lipids ratio in Large HDL | % | 23562 | 23559 |
| Free cholesterol to total lipids ratio in Large HDL | % | 23563 | 23559 |
| Triglycerides to total lipids ratio in Large HDL | % | 23564 | 23559 |
| Phospholipids to total lipids ratio in Medium HDL | % | 23567 | 23566 |
| Cholesterol to total lipids ratio in Medium HDL | % | 23568 | 23566 |
| Cholesteryl esters to total lipids ratio in Medium HDL | % | 23569 | 23566 |
| Free cholesterol to total lipids ratio in Medium HDL | % | 23570 | 23566 |
| Triglycerides to total lipids ratio in Medium HDL | % | 23571 | 23566 |
| Phospholipids to total lipids ratio in Small HDL | % | 23574 | 23573 |
| Cholesterol to total lipids ratio in Small HDL | % | 23575 | 23573 |
| Cholesteryl esters to total lipids ratio in Small HDL | % | 23576 | 23573 |
| Free cholesterol to total lipids ratio in Small HDL | % | 23577 | 23573 |
| Triglycerides to total lipids ratio in Small HDL | % | 23578 | 23573 |

## Table S3. ICD and OPCS codes for identifying incidents vascular and ocular outcomes.

| **Incident events** | **ICD-9** | **ICD-10** | **OPCS4** |
| --- | --- | --- | --- |
| Stroke | 433–435; 430–432 | I65, I66; I60–I63 |  |
| Heart failure | 425, 428 | I42, I50 |  |
| Myocardial infarction | 410, 411, 412 | I21, I22, I23, I24.1, I25.2 |  |
| Coronary heart disease | 410–414 | I20–I25 | K40–K46, K49, K50, K75 |
| Age-related macular disease | 3625 | H353 |  |
| Cataract | 366 | H25, H26, H28 |  |
| Glaucoma | 365 | H40 |  |
| Diabetic retinopathy | 362.01–362.07; 379.23, 362.81,361.0, 361.8, 361.9, 362.53, 369 | H36.0, E09.319, E08.319, E13.319, E10.319, E11.319 | C89x, C82x, C79x |

Mortality data were obtained from death registries issued by the NHS Information Centre (England and Wales). Cardiovascular disease (CVD) related mortality was determined by codes I00 to I09, I11, I13, and I20 to I51 (diseases of heart) and I60 to I69 (cerebrovascular diseases). The follow-up time was considered as the date of baseline assessment until April 28, 2021.

## Table S4. Baseline characteristics of included participants by dataset.

|  | **All (n=89,830)** | **Dataset by 1:1 randomly splitting** | |
| --- | --- | --- | --- |
|  |  | **Derivative set (n=44,915)** | **Applicative set (n=44,915)** |
| Age, years | 55.7 ± 8.1 | 55.7 ± 8.1 | 55.6 ± 8.1 |
| Female | 48,192 (53.7%) | 24,006 (53.5%) | 24,186 (53.9%) |
| White ethnicity | 84,689 (94.3%) | 42,382 (94.4%) | 42,307 (94.2%) |
| Townsend deprivation index | -1.4 ± 3.1 | -1.4 ± 3.1 | -1.4 ± 3.1 |
| College/university or above | 29,691 (33.0%) | 14,797 (32.9%) | 14,894 (33.2%) |
| Body mass index, kg/m^2^ | 27.4 ± 4.7 | 27.4 ± 4.7 | 27.4 ± 4.6 |
| Waist hip ratio | 0.9 ± 0.1 | 0.9 ± 0.1 | 0.9 ± 0.1 |
| SBP, mmHg | 137.4 ± 18.4 | 137.4 ± 18.5 | 137.4 ± 18.3 |
| Former/Current Smoker | 39,380 (43.8%) | 19,687 (43.8%) | 19,693 (43.9%) |
| Former/Current Alcohol intake | 85,859 (95.6%) | 42,979 (95.7%) | 42,880 (95.5%) |
| Physical activity, MET/min/week | 1,787 (816–3,573) | 1,794 (822–3,622) | 1,782 (813–3,546) |
| Total cholesterol, mmol/L | 5.7 ± 1.1 | 5.4 ± 0.6 | 5.4 ± 0.6 |
| HDL, mmol/L | 1.4 ± 0.4 | 1.4 ± 0.4 | 1.5 ± 0.4 |
| HbA1c levels, (%) | 5.4 ± 0.6 | 5.4 ± 0.6 | 5.4 ± 0.6 |
| Serum creatinine, mg/dL | 0.8 ± 0.2 | 0.8 ± 0.2 | 0.8 ± 0.2 |
| Hypertension | 64,502 (71.8%) | 32,222 (71.7%) | 32,280 (71.9%) |
| Diabetes mellitus | 4,524 (5.0%) | 2,212 (5.0%) | 2,312 (5.2%) |
| Antihypertensive drugs | 7,593 (8.5%) | 3,778 (8.4%) | 3,815 (8.5%) |
| Use of statin | 5,270 (5.9%) | 2,607 (5.8%) | 2,663 (5.9%) |
| Diet score | 3.9 ± 1.4 | 3.9 ± 1.4 | 3.9 ± 1.4 |

Data are presented as mean ± SD, No. (%), or median (IQR). SBP= systolic blood pressure; HDL= high-density lipoprotein; MET= metabolic equivalent of task.

## Table S5. Incident events and number in the application set.

|  | **All** | **Groups defined by metabolite-defined obesity** | | | | |
| --- | --- | --- | --- | --- | --- | --- |
|  |  | **NW** | **OW** | **OB** | **OE** | **UE** |
| No. of Subjects | 44,915 (100%) | 7,614 (16.9%) | 25,574 (56.9%) | 4,921 (11.0%) | 2,631 (5.9%) | 4,175 (9.3%) |
| **No. of events** |  |  |  |  |  |  |
| All-cause mortality | 2,233 (5.0%) | 204 (2.7%) | 1,135 (4.4%) | 424 (8.6%) | 227 (8.6%) | 243 (5.8%) |
| CVD-cause mortality | 535 (1.2%) | 30 (0.4%) | 253 (1.0%) | 117 (2.4%) | 57 (2.2%) | 78 (1.9%) |
| Stroke | 469 (1.0%) | 49 (0.6%) | 255 (1.0%) | 67 (1.4%) | 36 (1.4%) | 62 (1.5%) |
| Heart failure | 900 (2.0%) | 47 (0.6%) | 432 (1.7%) | 195 (4.0%) | 58 (2.2%) | 168 (4.0%) |
| Myocardial infarction | 911 (2.0%) | 40 (0.5%) | 477 (1.9%) | 217 (4.4%) | 69 (2.6%) | 108 (2.6%) |
| Coronary heart disease | 3,239 (7.2%) | 199 (2.6%) | 1,793 (7.0%) | 655 (13.3%) | 218 (8.3%) | 374 (9.0%) |
| AMD | 496 (1.1%) | 57 (0.8%) | 273 (1.1%) | 76 (1.5%) | 43 (1.6%) | 47 (1.1%) |
| Cataract | 3,281 (7.3%) | 426 (5.6%) | 1,821 (7.1%) | 498 (10.1%) | 218 (8.3%) | 318 (7.6%) |
| Glaucoma | 808 (1.8%) | 110 (1.4%) | 474 (1.9%) | 99 (2.0%) | 59 (2.2%) | 66 (1.6%) |
| Diabetic retinopathy | 532 (1.2%) | 57 (0.8%) | 247 (1.0%) | 110 (2.2%) | 41 (1.6%) | 77 (1.8%) |

Abbreviation: NW= normal weight of metabolic BMI; OW= overweight of metabolic BMI; OB= obesity of metabolic BMI; OE= metabolome-defined BMI over than actual BMI; UE= metabolome-defined BMI under than actual BMI; CVD= cardiovascular disease; AMD= age-related macular degeneration.

## Table S6. Subgroup analysis by sex of associations between overestimated metabolic BMI and incident endpoints in application set.

| **Incident events (OE vs. NW)** | **Female** | | **Male** | |
| --- | --- | --- | --- | --- |
|  | **HR (95% CI)** | **P*** | **HR (95% CI)** | **P*** |
| All-cause mortality | 1.90 (1.09, 3.30) | **0.023** | 1.49 (0.90, 2.49) | 0.124 |
| CVD-cause mortality | 2.30 (0.59, 8.91) | 0.085 | 4.51 (1.28, 15.83) | **0.019** |
| Stroke | 1.02 (0.28, 3.71) | 0.979 | 1.09 (0.39, 3.04) | 0.865 |
| Heart failure | 5.63 (1.68, 18.91) | **0.005** | 0.95 (0.35, 2.54) | 0.910 |
| Myocardial infarction | 2.11 (0.63, 7.10) | 0.229 | 2.92 (1.12, 7.57) | **0.028** |
| Coronary heart disease | 1.74 (1.02, 2.98) | **0.042** | 1.52 (0.93, 2.48) | 0.094 |
| AMD | 1.38 (0.59, 3.21) | 0.456 | 2.69 (0.70, 10.26) | 0.149 |
| Cataract | 1.09 (0.75, 1.58) | 0.651 | 1.11 (0.65, 1.90) | 0.693 |
| Glaucoma | 0.74 (0.35, 1.56) | 0.429 | 1.29 (0.54, 3.06) | 0.568 |
| Diabetic retinopathy | 1.67 (0.61, 4.57) | 0.315 | 1.12 (0.41, 3.10) | 0.823 |

OE=metabolome-defined BMI over than actual BMI; NW= normal weight of metabolic BMI; CVD= cardiovascular diseases; AMD= age-related macular degeneration; HR= hazard ratio; CI= confidence interval.

*Cox models adjusted for age (continuous), ethnicity (white/others), Townsend deprivation index (continuous), educational attainment (above or below college/university degree), actual body mass index (continuous), systolic blood pressure (continuous); smoking and alcohol drinking status (never/previous/present), physical activity (continuous), total cholesterol (continuous), high-density lipoprotein (continuous), hemoglobin A1c (continuous), serum creatinine (continuous), antihypertensive medications (yes/no), statin medications (yes/no), and healthy diet score (continuous).

## Table S7. Subgroup analysis by diet score of associations between overestimated metabolic BMI and incident endpoints in application set.

|  | **Healthy Diet Score (0–3 scores)** | | **Healthy Diet Score (4–7 scores)** | |
| --- | --- | --- | --- | --- |
| **Incident events (OE vs. NW)** | **HR (95% CI)** | **P*** | **HR (95% CI)** | **P*** |
| All-cause mortality | 2.48 (1.34, 4.59) | **0.004** | 1.55 (0.96, 2.50) | 0.073 |
| CVD-cause mortality | 5.46 (1.24, 23.94) | **0.024** | 3.38 (1.04, 10.97) | **0.043** |
| Stroke | 1.04 (0.31, 3.49) | 0.956 | 1.08 (0.37, 3.13) | 0.885 |
| Heart failure | 2.50 (0.84, 7.42) | 0.099 | 2.04 (0.73, 5.69) | 0.174 |
| Myocardial infarction | 8.96 (1.95, 41.08) | **0.005** | 1.42 (0.57, 3.55) | 0.453 |
| Coronary heart disease | 1.94 (1.01, 3.71) | **0.047** | 1.57 (1.01, 2.44) | **0.047** |
| AMD | 2.95 (0.67, 13.03) | 0.153 | 1.82 (0.83, 3.99) | 0.133 |
| Cataract | 1.28 (0.73, 2.26) | 0.389 | 1.12 (0.79, 1.59) | 0.532 |
| Glaucoma | 0.87 (0.33, 2.28) | 0.773 | 1.05 (0.55, 2.03) | 0.881 |
| Diabetic retinopathy | 1.68 (0.51, 5.58) | 0.394 | 1.10 (0.44, 2.76) | 0.832 |

OE= metabolome-defined BMI over than actual BMI; NW= normal weight of metabolic BMI; CVD= cardiovascular diseases; AMD= age-related macular degeneration; HR= hazard ratio; CI= confidence interval.

An unhealthy diet mode is considered with healthy diet score of 0–3, and a healthy diet mode is considered with healthy diet score of 4–7.

*Cox models adjusted for age (continuous), sex (female/male), ethnicity (white/others), Townsend deprivation index (continuous), educational attainment (above or below college/university degree), actual body mass index (continuous), systolic blood pressure (continuous); smoking and alcohol drinking status (never/previous/present), physical activity (continuous), total cholesterol (continuous), high-density lipoprotein (continuous), hemoglobin A1c (continuous), serum creatinine (continuous), antihypertensive medications (yes/no), and statin medications (yes/no).

## Table S8. Baseline characteristics of the included participants in the WHR model applicative set.

| **Characteristics** | **Participants in Module-I** | | | **Participants in Module-II** | | |
| --- | --- | --- | --- | --- | --- | --- |
|  | **NW_pre** | **OE_pre** | **P** | **OB_pre** | **UE_pre** | **P** |
| No. of subjects | 20,555 | 2,389 | - | 19,177 | 2,794 | - |
| **Waist-to-hip ratio (WHR)** |  |  |  |  |  |  |
| Actual WHR | 0.83 ± 0.07 | 0.75 ± 0.06 | <0.001 | 0.91 ± 0.06 | 1.00 ± 0.07 | <0.001 |
| Metabolomic-estimated | 0.82 ± 0.04 | 0.88 ± 0.06 | <0.001 | 0.92 ± 0.04 | 0.87 ± 0.06 | <0.001 |
| Body mass index, kg/m² | 25.3 ± 3.6 | 26.8 ± 5.3 | <0.001 | 29.4 ± 4.5 | 29.9 ± 5.0 | <0.001 |
| Systolic blood pressure, mm Hg | 134.2 ± 18.5 | 133.3 ± 18.7 | 0.025 | 140.6 ± 17.5 | 142.8 ± 18.3 | <0.001 |
| Total cholesterol, mmol/L | 5.8 ± 1.0 | 5.6 ± 1.1 | <0.001 | 5.7 ± 1.2 | 5.6 ± 1.1 | 0.011 |
| Hemoglobin A1c (HbA1c), % | 5.3 ± 0.4 | 5.4 ± 0.6 | <0.001 | 5.5 ± 0.7 | 5.5 ± 0.7 | 0.712 |
| Serum creatinine, mg/dL | 0.8 ± 0.1 | 0.8 ± 0.2 | <0.001 | 0.9 ± 0.2 | 0.8 ± 0.2 | <0.001 |
| Presence of hypertension, % | 12,820 (62.4%) | 1,499 (62.8%) | 0.719 | 15,633 (81.5%) | 2,329 (83.4%) | 0.019 |
| Presence of diabetes mellitus, % | 327 (1.6%) | 107 (4.5%) | <0.001 | 1,584 (8.3%) | 256 (9.2%) | 0.144 |
| Healthy diet score | 4.1 ± 1.4 | 4.1 ± 1.4 | 0.103 | 3.6 ± 1.4 | 3.6 ± 1.5 | 0.422 |

Data are presented as mean ± SD or No. (%).

WHR= waist-to-hip ratio; NW_pre= normal of metabolic WHR, OB_pre= obesity of metabolic WHR; OE_pre= metabolome-defined WHR over than actual WHR; UE_pre= metabolome-defined WHR under than actual WHR.

Normal-metWHR and obese-metWHR are defined based on normally prediction within a margin of error of -0.10 to +0.10 (central obesity is defined of WHR ≥ 0.85 for female and ≥0.90 for male); OE-metWHR is defined of metWHR-actWHR >0.1; and UE-metWHR is defined of metWHR-actWHR < -0.1.

P-value was estimated based on chi-squared or independent t-test, where appropriate.

## Table S9. Associations of metabolome WHR phenotypes with risks of cardiovascular and ocular disease outcomes in the applicative set.

|  | **Model 1 *** | | **Model 2 †** | |
| --- | --- | --- | --- | --- |
|  | **HR (95%CI)** | **P** | **HR (95%CI)** | **P** |
| **OE_pre vs. NW_pre** |  |  |  |  |
| All-cause mortality | 1.20 (0.95, 1.50) | 0.122 | 1.07 (0.79, 1.46) | 0.662 |
| CVD-cause mortality | 2.32 (1.55, 3.47) | **<0.001** | 2.05 (1.11, 3.79) | **0.022** |
| Stroke | 1.50 (0.98, 2.30) | 0.063 | 1.27 (0.68, 2.38) | 0.452 |
| Heart failure | 1.90 (1.36, 2.67) | **<0.001** | 1.48 (0.87, 2.53) | 0.149 |
| Myocardial infarction | 2.42 (1.70, 3.44) | **<0.001** | 2.48 (1.45, 4.24) | **0.001** |
| Coronary heart disease | 1.59 (1.31, 1.92) | **<0.001** | 1.69 (1.29, 2.20) | **<0.001** |
| AMD | 1.11 (0.74, 1.66) | 0.610 | 0.98 (0.58, 1.68) | 0.950 |
| Cataract | 1.14 (0.97, 1.33) | 0.125 | 1.12 (0.90, 1.40) | 0.317 |
| Glaucoma | 0.87 (0.60, 1.26) | 0.467 | 0.83 (0.51, 1.34) | 0.446 |
| Diabetic retinopathy | 1.24 (0.80, 1.93) | 0.335 | 0.74 (0.39, 1.40) | 0.359 |
| **UE_pre vs. OB_pre** |  |  |  |  |
| All-cause mortality | 1.20 (1.05, 1.38) | **0.009** | 1.04 (0.85, 1.26) | 0.707 |
| CVD-cause mortality | 1.25 (0.96, 1.63) | 0.101 | 0.90 (0.60, 1.33) | 0.583 |
| Stroke | 1.29 (0.95, 1.76) | 0.109 | 1.07 (0.68, 1.69) | 0.768 |
| Heart failure | 1.38 (1.13, 1.68) | **0.002** | 1.02 (0.76, 1.36) | 0.902 |
| Myocardial infarction | 1.07 (0.86, 1.31) | 0.554 | 0.85 (0.64, 1.14) | 0.284 |
| Coronary heart disease | 0.97 (0.86, 1.09) | 0.631 | 0.79 (0.67, 0.94) | **0.006** |
| AMD | 1.45 (1.05, 2.02) | **0.026** | 1.08 (0.69, 1.70) | 0.742 |
| Cataract | 1.14 (1.00, 1.30) | **0.044** | 1.06 (0.89, 1.28) | 0.502 |
| Glaucoma | 1.20 (0.93, 1.55) | 0.172 | 1.14 (0.79, 1.64) | 0.491 |
| Diabetic retinopathy | 1.05 (0.78, 1.41) | 0.762 | 0.73 (0.48, 1.12) | 0.152 |

WHR= waist-to-hip ratio; NW_pre= normal of metabolic WHR, OB_pre= obesity of metabolic WHR; OE_pre= metabolome-defined WHR over than actual WHR; UE_pre= metabolome-defined WHR under than actual WHR; CVD= cardiovascular diseases; AMD= age-related macular degeneration; HR= hazard ratio; CI= confidence interval.

* Model 1: adjusted for age (continuous), sex (female and male), ethnicity (white/others), and Townsend deprivation index (continuous).

† Model 2: further adjusted for educational attainment (above or below college/university degree), actual waist-to-hip ratio (continuous), systolic blood pressure (continuous); smoking and alcohol drinking status (never/previous/present), physical activity (continuous), total cholesterol (continuous), hemoglobin A1c (continuous), serum creatinine (continuous), and healthy diet score (continuous).

## Table S10. List summarizing metabolic obesity fingerprints identified in the GDES cohort using LC/MS profiling.

| **Compounds** | **Group** | **Subgroup** | **β** | **95% CI** | **P-value** |
| --- | --- | --- | --- | --- | --- |
| LPC(16:0/0:0) | GP | LPC | -0.693 | (-1.097, -0.288) | 0.001 |
| LPC(15:0/0:0) | GP | LPC | -0.635 | (-1.043, -0.227) | 0.002 |
| 2-Hexadecanoylthio-1-ethylphosphorylcholine | FA | Others | -0.606 | (-1.010, -0.203) | 0.003 |
| LPC(0:0/15:0) | GP | LPC | -0.602 | (-1.007, -0.198) | 0.003 |
| LPC(O-16:1) | GP | LPC-O | -0.593 | (-0.968, -0.218) | 0.002 |
| LPC(0:0/16:0) | GP | LPC | -0.587 | (-0.992, -0.182) | 0.005 |
| PAF C-18:1 | GP | LPC | -0.572 | (-0.977, -0.167) | 0.006 |
| LPC(0:0/20:1) | GP | LPC | -0.566 | (-0.969, -0.163) | 0.006 |
| LPC(17:1/0:0) | GP | LPC | -0.556 | (-0.990, -0.121) | 0.012 |
| LPE(O-17:1) | GP | LPE | -0.553 | (-0.935, -0.171) | 0.005 |
| LPE(P-18:0) | GP | LPE-P | -0.549 | (-0.923, -0.175) | 0.004 |
| LPC(O-14:0) | GP | LPC-O | -0.549 | (-1.080, -0.018) | 0.043 |
| LPC(0:0/18:0) | GP | LPC | -0.549 | (-1.002, -0.095) | 0.018 |
| LPE(P-17:0) | GP | LPE-P | -0.542 | (-0.927, -0.158) | 0.006 |
| LPA(22:6) | GP | LPA | -0.523 | (-0.877, -0.170) | 0.004 |
| LPC(20:1/0:0) | GP | LPC | -0.515 | (-0.909, -0.121) | 0.010 |
| 20,26-dihydroxyecdysone | Hormones and hormone related compounds | Hormones and hormone related compounds | -0.510 | (-0.867, -0.152) | 0.005 |
| LPC(18:0/0:0) | GP | LPC | -0.507 | (-0.899, -0.115) | 0.011 |
| LPE(0:0/20:1) | GP | LPE | -0.502 | (-0.928, -0.076) | 0.021 |
| Carnitine C22:1 | FA | CAR | -0.490 | (-0.875, -0.106) | 0.012 |
| LPA(20:4) | GP | LPA | -0.471 | (-0.865, -0.077) | 0.019 |
| LPC(16:2/0:0) | GP | LPC | -0.466 | (-0.918, -0.015) | 0.043 |
| LPC(20:4/0:0) | GP | LPC | -0.457 | (-0.844, -0.070) | 0.021 |
| LPE(P-16:0) | GP | LPE-P | -0.449 | (-0.812, -0.085) | 0.016 |
| LPE(O-18:1/0:0) | GP | LPE | -0.439 | (-0.797, -0.081) | 0.016 |
| LPE(0:0/16:0) | GP | LPE | -0.430 | (-0.794, -0.067) | 0.020 |
| LPC(0:0/20:4) | GP | LPC | -0.428 | (-0.809, -0.047) | 0.028 |
| LPE(0:0/20:4) | GP | LPE | -0.419 | (-0.798, -0.041) | 0.030 |
| LPC(16:1/0:0) | GP | LPC | -0.412 | (-0.790, -0.033) | 0.033 |
| LPC(19:1/0:0) | GP | LPC | -0.411 | (-0.766, -0.056) | 0.023 |
| LPC(O-18:1) | GP | LPC-O | -0.411 | (-0.788, -0.034) | 0.033 |
| LPG(22:5) | GP | LPG | -0.407 | (-0.796, -0.018) | 0.040 |
| LPE(20:1/0:0) | GP | LPE | -0.403 | (-0.801, -0.005) | 0.047 |
| LPC(0:0/16:1) | GP | LPC | -0.399 | (-0.774, -0.023) | 0.037 |
| LPC(19:2) | GP | LPC | -0.385 | (-0.734, -0.036) | 0.031 |
| LPG(16:0) | GP | LPG | -0.382 | (-0.728, -0.035) | 0.031 |
| LPE(P-18:1) | GP | LPE-P | -0.381 | (-0.749, -0.013) | 0.042 |
| 10-deoxyformamycin | Others | Others | -0.380 | (-0.721, -0.040) | 0.028 |
| LPC(18:2/0:0) | GP | LPC | -0.379 | (-0.746, -0.011) | 0.043 |
| LPE(16:0/0:0) | GP | LPE | -0.371 | (-0.723, -0.019) | 0.039 |
| 2-Thio-PAF | FA | Others | -0.367 | (-0.707, -0.028) | 0.034 |
| LPS(20:0) | GP | LPS | -0.364 | (-0.714, -0.014) | 0.042 |
| Ser-Arg | Amino acid and Its metabolites | Small Peptide | -0.361 | (-0.713, -0.010) | 0.044 |
| Phe-Ala-Ser | Amino acid and Its metabolites | Small Peptide | -0.359 | (-0.686, -0.033) | 0.031 |
| 1-O-Hexadecyl-2-O-ethyl-sn-glycero-3-phosphocholine | Others | Others | -0.357 | (-0.711, -0.004) | 0.048 |
| 2-Hydroxyhexanoic acid | Organic acid and Its derivatives | Organic acid and Its derivatives | 0.273 | (0.008, 0.539) | 0.044 |
| Isochodeoxycholic acid | Bile acids | Bile acids | 0.335 | (0.006, 0.665) | 0.046 |
| Hydroxyurea | Others | Others | 0.336 | (0.015, 0.656) | 0.040 |
| S1P(d18:2) | SL | SPH | 0.343 | (0.008, 0.679) | 0.045 |
| Cys-Pro | Amino acid and Its metabolites | Small Peptide | 0.349 | (0.026, 0.671) | 0.034 |
| 12-ketolithocholic acid | Bile acids | Bile acids | 0.355 | (0.094, 0.616) | 0.008 |
| FFA(18:2) | FA | FFA | 0.355 | (0.010, 0.700) | 0.044 |
| Guanidine | Alcohol and amines | Polyamines | 0.371 | (0.028, 0.713) | 0.034 |
| L-Serine | Amino acid and Its metabolites | Amino acids | 0.383 | (0.075, 0.691) | 0.015 |
| Arg-Phe-Val-Asp | Amino acid and Its metabolites | Small Peptide | 0.383 | (0.040, 0.725) | 0.029 |
| 5'-Deoxy-5'-(Methylthio) Adenosine | Nucleotide and Its metabolites | Nucleotide and Its metabolites | 0.394 | (0.030, 0.757) | 0.034 |
| Phenyllactate(Pla) | Organic acid and Its derivatives | Organic acid and Its derivatives | 0.409 | (0.099, 0.718) | 0.010 |
| L-3-Phenyllactic acid | Organic acid and Its derivatives | Organic acid and Its derivatives | 0.409 | (0.100, 0.718) | 0.009 |
| α-Hydroxyglutaric Acid (sodium salt) | Organic acid and Its derivatives | Organic acid and Its derivatives | 0.418 | (0.074, 0.762) | 0.017 |
| Ac-YVAD-CMK | Amino acid and Its metabolites | Small Peptide | 0.472 | (0.136, 0.808) | 0.006 |
| Hydroferulic acid | Organic acid and Its derivatives | Organic acid and Its derivatives | 0.513 | (0.162, 0.864) | 0.004 |
| Glu-Val | Amino acid and Its metabolites | Small Peptide | 0.555 | (0.169, 0.942) | 0.005 |
| Homovanillic Acid sulfate (sodium salt) | Organic acid and Its derivatives | Organic acid and Its derivatives | 0.558 | (0.112, 1.004) | 0.014 |
| Hydroxyphenyllactic acid | Organic acid and Its derivatives | Organic acid and Its derivatives | 0.597 | (0.138, 1.056) | 0.011 |

# SUPPLEMENTARY Method

## Method S1. Profiling details and quality control processes of 1H-NMR profiling in UKB.

Details about the metabolomic profiling protocol have been described elsewhere.^1^ Sample collection was undertaken at baseline in 22 local assessment centers across 241 the UK between 2007 and 2010. The blood sample handling and storage protocol has been previously described.^2^ The metabolomic profiling took place in Finland between 2019 and 2020 using six NMR spectrometers. Accredited quality control was done during the whole process to eliminate systemic and technical variance, and only samples and biomarkers that underwent the quality control process were stored in the UK Biobank dataset and used in our present study.

EDTA plasma samples from aliquot were prepared in 96-well plates by UK Biobank laboratory (Stockport, UK). TECAN freedom EVO 150 robotic liquid handlers were used for aliquoting plasma samples. The plasma samples were shipped in batches to Nightingale Health laboratories in Finland on dry ice. Before preparation, frozen samples were thawed slowly, mixed gently and centrifuged. Aliquots of each sample were mixed with a phosphate buffer and subjected to measurement using six 500 MHz NMR spectrometers. Nightingale Health’s proprietary software (quantification library 2020) was used to quantify the biomarkers.

Each 96-well plate included two internal control samples provided by Nightingale Health to track consistency across multiple spectrometers. Four sets of internal control samples with different biomarker concentration span were used across 1,352 96-well plates and interleaved between NMR instruments for extended periods. Blind duplicate samples from UK Biobank were included on each 96-well plate, with position revealed only after results delivery. The coefficient of variation 43 (CV) distributions for the blinded replicates and Nightingale Health’s internal control samples were typically below 5%. These results met pre-specified CV targets for each set of approximately 20,000 consecutively measured samples.

The NMR biomarker data in the UK Biobank can generally be used for epidemiological analyses without any preprocessing and can in principle be analyzed in the same manner as the clinical chemistry data available in UKB. Biomarker values substantially affected by interfering substances have been removed during the quality control procedures.

**References**

1. Julkunen, H., Cichońska, A., Tiainen, M., Koskela, H., Nybo, K., Mäkelä, V., Nokso-Koivisto, J., Kristiansson, K., Perola, M., Salomaa, V., Jousilahti, P., Lundqvist, A., Kangas, A. J., Soininen, P., Barrett, J. C., & Würtz, P. (2023). Atlas of plasma NMR biomarkers for health and disease in 118,461 individuals from the UK Biobank. Nature communications, 14(1), 604.

2. Elliott, P., Peakman, T. C., & UK Biobank (2008). The UK Biobank sample handling and storage protocol for the collection, processing and archiving of human blood and urine. International journal of epidemiology, 37(2), 234–244.

## Method S2. Assessment of diet.

In order to assess diet, we used UK Biobank (UKB) Food Frequency Questionnaire scores at baseline. For the current paper, we were interested in (1) UKB cooked vegetable intake (Field ID:1289; On average how many heaped tablespoons of cooked vegetables would you eat per day?), (2) UKB salad/raw vegetable intake (Field ID:1299; On average how many heaped tablespoons of raw vegetables would you eat per day?), (3) UKB fresh fruit intake (Field ID:1309; About how many pieces of fresh fruit would you eat per day?), (4) UKB dried fruit intake (Field ID:1319; About how many pieces of dried fruit would you eat per day?), (5) UKB oily fish intake (Field ID:1329; How often do you eat oily fish? (e.g. sardines, salmon, mackerel, herring); coded as never, less than once a week, once a week, 2-4 times a week, 5-6 times a week, once or more daily, do not know, and prefer not to answer), (6) UKB non-oily fish intake (Field ID:1339; How often do you eat other types of fish? (e.g. cod, tinned tuna, haddock); coded as never, less than once a week, once a week, 2-4 times a week, 5-6 times a week, once or more daily, do not know, and prefer not to answer), (7) UKB processed meat intake (Field ID:1349; How often do you eat processed meats (such as bacon, ham, sausages, meat pies, kebabs, burgers, chicken nuggets)?; coded as never, less than once a week, once a week, 2-4 times a week, 5-6 times a week, once or more daily, do not know, and prefer not to answer), (8) UKB poultry intake (Field ID:1359; How often do you eat chicken, turkey or other poultry? (Do not count processed meats); coded as never, less than once a week, once a week, 2-4 times a week, 5-6 times a week, once or more daily, do not know, and prefer not to answer), (9) UKB beef intake (Field ID:1369; How often do you eat beef? (Do not count processed meats); coded as never, less than once a week, once a week, 2- 4 times a week, 5-6 times a week, once or more daily, do not know, and prefer not to answer), (10) UKB lamb/mutton intake (Field ID:1379; How often do you eat lamb/mutton? (Do not count processed meats); coded as never, less than once a week, once a week, 2-4 times a week, 5-6 times a week, once or more daily, do not know, and prefer not to answer), (11) UKB pork intake (Field ID:1389; How often do you eat pork? (Do not count processed meats); coded as never, less than once a week, once a week, 2-4 times a week, 5-6 times a week, once or more daily, do not know, and prefer not to answer), (12) UKB bread intake (Field ID:1438; How many slices of bread do you eat each week?) and type (Field ID:1448; What type of bread do you mainly eat?; coded as white, brown, wholemeal or wholegrain, other type of bread, do not know, prefer not to answer), (13) UKB cereal intake (Field ID:1458; How many bowls of cereal do you eat a week?) and type (Field ID:1468), (14) UKB tea intake (Field ID:1488; How many cups of tea do you drink each day? (Include black and green tea), (15) UKB coffee intake (Field ID:1498; How many cups of coffee do you drink each day? (Include decaffeinated coffee)), and (16) UKB water intake (Field ID:1528; How many glasses of water do you drink each day?) data fields.

## Method S3. Profiling details and quality control processes of LC/MS profiling in the GDES cohort.

The sample stored at -80 °C refrigerator was thawed on ice and vortexed for 10 s. 50 μL of sample and 300 μL of extraction solution (ACN : Methanol = 1:4, V/V) containing internal standards were added into a 2 mL microcentrifuge tube. The sample was vortexed for 3 min and then centrifuged at 12000 rpm for 10 min (4 °C). 200 μL of the supernatant was collected and placed in -20 °C for 30 min, and then centrifuged at 12000 rpm for 3 min (4 °C). A 180 μL aliquots of supernatant were transferred for LC-MS analysis.

The sample extracts were analyzed using an LC-ESI-MS/MS system (UPLC, ExionLC 48 AD, https://sciex.com.cn/; MS, QTRAP® System, https://sciex.com/). The analytical conditions were as follows, UPLC: column, Waters ACQUITY UPLC HSS T3 C18 (1.8µm, 2.1 mm*100 mm); column temperature, 40 °C; flow rate, 0.4 mL/min; injection volume, 2μL; solvent system, water (0.1% formic acid): acetonitrile (0.1% formic acid); gradient program, 95:5 V/V at 0 min, 10:90 V/V at 11.0 min, 10:90 V/V at 12.0 min, 95:5 V/V at 12.1 min, 95:5 V/V at 14.0 min.

LIT and triple quadrupole (QQQ) scans were acquired on a triple quadrupole-linear ion trap mass spectrometer (QTRAP), QTRAP® LC-MS/MS System, equipped with an ESI Turbo Ion-Spray interface, operating in positive and negative ion mode, and controlled by Analyst 1.6.3 software (Sciex). The ESI source operation parameters were as follows: source temperature 500 °C; ion spray voltage (IS) 5500 V (positive), -4500 V (negative); ion source gas I (GSI), gas II (GSII), curtain gas (CUR) were set at 55, 60, and 25.0 psi, respectively; the collision gas (CAD) was high. Instrument tuning and mass calibration were performed with 10 and 100 μmol/L polypropylene glycol solutions in QQQ and LIT modes, respectively. A specific set of MRM transitions were monitored for each period according to the metabolites eluted within this period.

Quality control samples (QC) were prepared by mixing sample extracts to analyze the reproducibility of the samples under the same processing method. During the instrument analysis process, one QC sample was inserted every ten analytical samples to monitor the reproducibility of the analysis process. Overlapping analysis of the total ion chromatogram (TIC) of different QC samples were used to evaluate the reproducibility of metabolite extraction and detection, i.e., technical reproducibility. The high stability of the instrument provides important guarantees for the reproducibility and reliability of the data.

To monitor the presence of any residual substances during the detection process, blank samples were included at various stages of the experiment. The appearance 49 of peaks in these samples could indicate cross-contamination between samples or the presence of unwanted substances.

In addition, to ensure the reliability and accuracy of the experimental results, internal standards with known concentrations were added to the quality control process. The response of the internal standards was used as an indicator of the stability of the detection process, with smaller variation in response indicating higher data quality. The CVs for all internal standards in the current profiling were all below 0.01.

**Reference:**

1. Chen W, Gong L, Guo Z, et al. A Novel Integrated Method for Large-Scale Detection, Identification, and Quantification of Widely Targeted Metabolites: Application in the Study of Rice Metabolomics[J]. Molecular Plant, 2013, 6(6):1769-1780.

2. Fraga, C.G., et al., Signature-discovery approach for sample matching of a nerve-agent precursor using liquid chromatography-mass spectrometry, XCMS, and chemometrics. Anal Chem, 2010. 82(10): p. 4165-73.

3. Viant M R, Kurland I J, Jones M R, et al. How close are we to complete annotation of metabolomes? Current opinion in chemical biology, 2017, 36: 64-69.

## Method S4. Metabolome-based BMI and WHR models.

The values in metabolites dataset were standardized with z-score using the mean and SD per metabolite. Then, ridge modeling with ten-fold cross-validation were performed for the (unstandardized) log_e_-transformed BMI or WHR and each metabolites dataset. Training and testing (hold-out) sets were generated by splitting participants into ten sets with one set as a testing (hold-out) set and the remaining nine sets as a training set and iterating all combinations over those ten sets. that is, overfitting was controlled using ten-fold iteration with ten testing (hold-out) sets, and hyperparameter was decided using ten-fold cross-validation with internal training and validation sets from each training set. Consequently, this procedure generated ten fitted sparse models for metabolites and one single testing (hold-out) set-derived prediction from metabolites for each participant.

For the ridge-modeling iteration analysis, ten ridge models were repeatedly generated with the above modeling scheme. At the end of each iteration, the variable that was retained across ten models and that had the highest absolute value for the mean of ten β-coefficients was removed from the input metabolites.
